# Does Exposure to Ambient Air Pollution Affect Gestational Age and Newborn Weight?—A Systematic Review

**DOI:** 10.3390/healthcare12121176

**Published:** 2024-06-11

**Authors:** Bartlomiej Grabowski, Stepan Feduniw, Anna Orzel, Marcin Drab, Jan Modzelewski, Michal Pruc, Zuzanna Gaca, Lukasz Szarpak, Michal Rabijewski, Arkadiusz Baran, Anna Scholz

**Affiliations:** 1 Department of Urology, Military Institute of Medicine, Szaserow 128, 04-349 Warsaw, Poland; bgrabowski@wim.mil.pl; 2 Department of Gynecology, University Hospital Zürich, Frauenklinikstrasse 10, 8091 Zürich, Switzerland; 3 I Department of Obstetrics and Gynecology, Centre of Postgraduate Medical Education, 01-004 Warsaw, Poland; anna.k.orzel@gmail.com (A.O.); marcindrabmdm@gmail.com (M.D.); arkadiusz-baran@tlen.pl (A.B.); 4 Department of Reproductive Health, Centre of Postgraduate Medical Education, 01-813 Warsaw, Poland; jmodzelewski@cmkp.edu.pl (J.M.); mrabijewski@cmkp.edu.pl (M.R.); scholzannak@gmail.com (A.S.); 5 Research Unit, Polish Society of Disaster Medicine, 05-806 Warsaw, Poland; m.pruc@ptmk.org (M.P.); zuzanna.k.gaca@gmail.com (Z.G.); 6 Department of Public Health, International European University, 03187 Kyiv, Ukraine; 7 Department of Clinical Research and Development, LUXMED Group, 02-676 Warsaw, Poland; lukasz.szarpak@gmail.com; 8 Henry JN Taub Department of Emergency Medicine, Baylor College of Medicine, Houston, TX 77030, USA

**Keywords:** small for gestational age (SGA), term low birth weight (TLBW), ambient air pollution, particulate matter ≤ 10 μm (PM_10_), particulate matter ≤ 2.5 μm (PM_2.5_), ozone (O_3_), sulfur dioxide (SO_2_), carbon monoxide (CO), nitrogen dioxide (NO_2_)

## Abstract

Current evidence suggests that airborne pollutants have a detrimental effect on fetal growth through the emergence of small for gestational age (SGA) or term low birth weight (TLBW). The study’s objective was to critically evaluate the available literature on the association between environmental pollution and the incidence of SGA or TLBW occurrence. A comprehensive literature search was conducted across Pubmed/MEDLINE, Web of Science, Cochrane Library, EMBASE, and Google Scholar using predefined inclusion and exclusion criteria. The methodology adhered to the PRISMA guidelines. The systematic review protocol was registered in PROSPERO with ID number: CRD42022329624. As a result, 69 selected papers described the influence of environmental pollutants on SGA and TLBW occurrence with an Odds Ratios (ORs) of 1.138 for particulate matter ≤ 10 μm (PM_10_), 1.338 for particulate matter ≤ 2.5 μm (PM_2.5_), 1.173 for ozone (O_3_), 1.287 for sulfur dioxide (SO_2_), and 1.226 for carbon monoxide (CO). All eight studies analyzed validated that exposure to volatile organic compounds (VOCs) is a risk factor for SGA or TLBW. Pregnant women in the high-risk group of SGA occurrence, i.e., those living in urban areas or close to sources of pollution, are at an increased risk of complications. Understanding the exact exposure time of pregnant women could help improve prenatal care and timely intervention for fetuses with SGA. Nevertheless, the pervasive air pollution underscored in our findings suggests a pressing need for adaptive measures in everyday life to mitigate worldwide environmental pollution.

## 1. Introduction

Intra-uterine growth is a crucial indicator reflecting the well-being of the fetus. Therefore, fetal growth abnormalities could arise from various pregnancy-related complications and are directly linked to increased fetal mortality [1]. According to the Royal College of Obstetricians and Gynaecologists guidelines, a newborn is considered small for gestational age (SGA) if the birth weight is below the 10th percentile based on customized growth charts [2,3,4]. It is estimated that while the majority of hypotrophic infants fall under the SGA definition, about 50–70% are constitutionally small but otherwise healthy newborns with growth aligned with parental metrics [5].

Conversely, the term low birth weight (TLBW) refers to an infant with a birth weight < 2500 g [6]. Historically, TLBW was widely used as an indicator to assess infant well-being, influencing subsequent clinical decisions. At the same time, SGA is a more precise term, reflecting the underlying pathology, not only lower neonatal weight. The SGA diagnosis could be made if the estimated fetal weight falls below the 10th percentile [5,7,8]. The exact pathomechanism underlying SGA is not fully understood. There are several contributing factors, including maternal chronic conditions, fetal abnormalities, and those related to placental dysfunction [3,8]. Maternal exposure to environmental factors, including exposure to medications, residential building materials, and tobacco, significantly increases the risks of adverse outcomes such as preterm delivery, spontaneous abortion, growth restriction, and other postnatal complications [9,10,11]. Exogenous substances from the maternal diet and air quality also significantly affect fetal well-being.

It should be noted that environmental factors are challenging to evaluate because of individual preferences, varying environments, and differing socioeconomic conditions. According to the World Health Organization (WHO), air pollution has emerged as the single biggest environmental threat to human health, estimated to cause 7 million premature deaths annually, making it the fourth most significant risk factor for early death globally in 2019, preceded only by hypertension, tobacco use, and poor diet [12,13]. Airborne pollutants that have been identified as responsible for multifactorial damage to the body include particulate matter ≤ 10 μm (PM_10_) and particulate matter ≤ 2.5 μm (PM_2.5_), ozone (O_3_), carbon monoxide (CO), NO_x_ as combined nitric oxide (NO) and nitrogen dioxide (NO_2_), sulfur dioxide (SO_2_), and volatile organic compounds (VOCs) or other less explored pollutants. Most anthropogenic air pollutants enter the atmosphere as a consequence of contemporary industrialization and urbanization, the combustion of fossil fuels in thermal power plants, industrial production, and transportation vehicles, known as traffic-related air pollutants (TRAPs) [13,14,15,16]. While the adverse health effects of these substances have already been thoroughly researched in the adult population, more knowledge is still needed about their impact on health during the prenatal period. Gaining a comprehensive understanding of the precise effects of prenatal exposure to these environmental contaminants is crucial. Acquiring such knowledge is important for creating accurate public health interventions and establishing strong policy frameworks that aim to safeguard the health of pregnant women and their developing fetuses. This disparity is especially noticeable due to the susceptibility of the developing fetus to environmental stressors.

Past reviews have confirmed the overall effect of ambient air pollution on health outcomes [15,16]. There is a lack of systematic reviews that thoroughly analyze and study the impacts of certain air pollutants, mainly because it is difficult to isolate and evaluate the influence of individual pollutants on fetal development [17]. Existing assessments have shown a simultaneous impact on cases of preterm delivery and fetal growth restriction, the latter being a condition where the baby is naturally smaller if born prematurely [18,19]. This overlap complicates the study and presents a potential for severe confounding bias. To fill this important knowledge vacuum, we conducted a systematic review specifically on fetal growth restriction at term, known as SGA or TLGW.

The aim of this study was to determine the environmental factors that pose the highest risk for unfavorable fetal development outcomes throughout pregnancy. This systematic review focuses on analyzing the complex relationships between previously reported findings on the impact of various environmental pollutants, such as PM_10_, PM_2.5_, O_3_, CO, NO_x_, and SO_2_, on the occurrence of SGA or TLBW in infants. We aimed to analyze the existing research and pinpoint key areas that require additional investigation to strengthen the body of information that supports the development of effective public health interventions.

## 2. Materials and Methods

The current systematic review was conducted in adherence to the Preferred Reporting Items for Systematic Reviews and Meta-Analyses (PRISMA) guidelines [20]. The systematic review protocol was registered in PROSPERO under the ID number: CRD42022329624.

A comprehensive literature search was performed across databases, including Pubmed/MEDLINE, Web of Science, Cochrane Library, EMBASE, and Google Scholar, using the search strategy presented in Table 1.

All searches were conducted on 1 August 2023 and confined to articles in English, German, or Polish without any restrictions to the publication date. Additionally, the references of all the included studies were hand-searched for any additional relevant articles.

All types of evaluative study designs were included and assessed. Two reviewers (SF and BG) independently screened the studies based on the title, abstract, and full text. Studies that met the selection criteria were included. The reference lists of the included studies underwent additional screening. Each included study was assessed on a scale (0 = not relevant, 1 = possibly relevant, and 2 = very relevant). Only studies that scored at least 1 point were included in the analysis. Any disagreements between reviewers were resolved by the third researcher (AK).

The PI(E)CO question was “Does exposure to ambient air pollution influence the risk of small-for-gestational-age?”. The Population (P) comprised pregnant women exposed to ambient air pollution. The Exposure (E) was various ambient air pollution (PM_2.5_—particle matter ≤ 2.5 µm, PM_10_—particle matter ≤ 10 µm, CO—carbon monoxide, VOC—volatile organic compounds, NO_x_—nitrogen dioxide, SO_2_—sulfur dioxide, ground-level O_3_—ozone). Studies, including air concentrations of individual heavy metals as a compartment of particle matter pollution, were not included, as the information was not reported in standard pollution measurements. As the included studies were mostly retrospective, none of them adjusted for specific information about the concentration of molecules, including particulate matters (PMs). Exposure had to last at least three months during pregnancy, excluding indoor and natural sources of pollution. The Comparative group (C) consisted of pregnant women either not exposed to ambient air pollution or minimally exposed to ambient air pollutants (values in the first quartile). The study populations were compared to historic cohorts or cohorts from healthy environments, as defined by the authors. The outcome (O) was the occurrence of SGA (as defined by the authors of included studies) or TLBW < 2500 g. Studies (S) included in the analyses were either retrospective or prospective, with a control group of unaffected or minimally affected pregnant women.

Due to the large amplitude of air pollution values, the exposed and unexposed groups differ between the studies. Despite different values used as the cut-off point for inclusion in the exposed group, authors in the studies always compared the exposed group (from the second to the fourth quartile) to the group considered unexposed (values in the first quartile). This distinction between the exposed and unexposed groups based on pollution thresholds allows for comparisons of the impact of air pollution within culturally, geographically, socially, and environmentally similar cohorts.

The risk of bias was assessed independently by two authors (SF and BG) using the Newcastle–Ottawa scale [21]. The third reviewer (JM) resolved any discrepancies in the selection process. Predominantly, the studies included were of moderate to high quality.

Due to the included studies’ heterogeneity, it was impossible to perform a quantitative synthesis. Nevertheless, a comprehensive comparison of the included studies is provided in the summary.

## 3. Results

**Figure 1:**
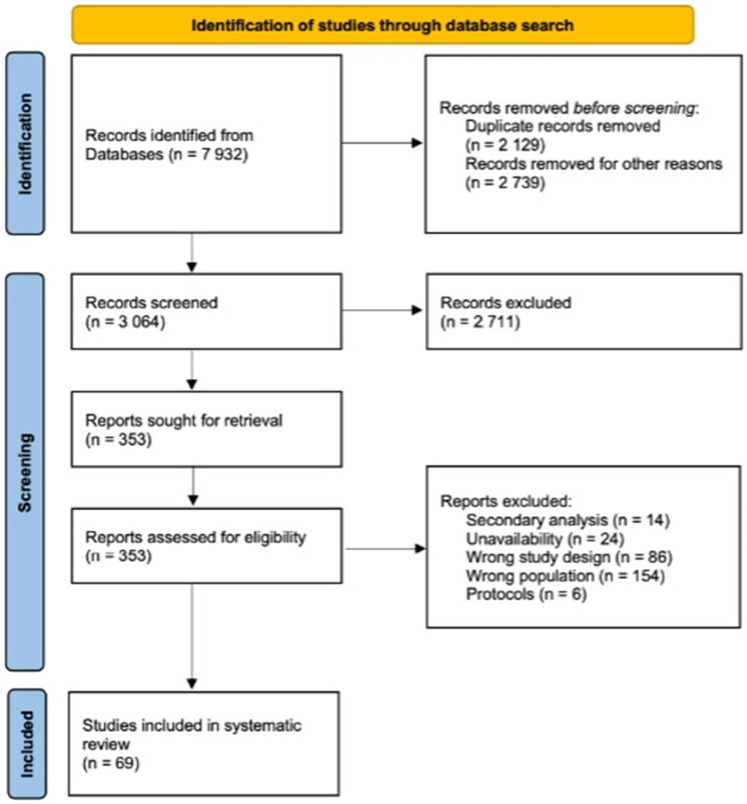
PRISMA systematic review flow diagram.

The study selection process is comprehensively presented in Figure 1, providing a flow diagram of the evaluation process. Initially, a total of 7932 articles were identified from the database search (Pubmed/MEDLINE = 1778, Web of Science = 2035, Cochrane Library = 138, EMBASE = 674, and Google Scholar = 3307). After removing duplicates, 3064 publications underwent preliminary evaluations based on their titles and abstracts. Just 353 articles were selected for full-text screening, as shown in Figure 1. The study was written according to guidelines, and the PRISMA checklist is published in Supplementary Table S1 [20].

The systematic review comprised a total of 69 papers [22,23,24,25,26,27,28,29,30,31,32,33,34,35,36,37,38,39,40,41,42,43,44,45,46,47,48,49,50,51,52,53,54,55,56,57,58,59,60,61,62,63,64,65,66,67,68,69,70,71,72,73,74,75,76,77,78,79,80,81,82,83,84,85,86,87,88,89,90]. Supplementary Table S2 shows the Newcastle–Ottawa risk bias score of the included studies.

Among these, 25 studies were from North America. Of these, 19 studies were conducted in the United States of America [29,41,43,44,45,53,55,58,60,63,64,66,67,78,84,85,87,88,90] and six studies in Canada [50,51,52,54,74,77].

There were 19 conducted in Asia: 11 in China [23,25,26,28,30,32,33,34,36,37,46], three in Korea [42,76,81], three in Taiwan [80,82,86], one in Israel [27], and one in Japan [69].

There were 16 studies performed throughout Europe: three in Lithuania [48,83,89], three in the Netherlands [38,65,70], three in the UK [35,57,61], two in Spain [22,72], two in Sweden [62,68], one in Norway [71], one in Poland [39], and one in Italy [49].

Six studies were conducted in South America: four in Brazil [47,56,59,73], one in Chile [31], and one in Peru [40]. Two studies were from the other parts of the world—one from Australia [75] and one from South Africa [24]. The selected studies encompassed aggregated data from 20,024,479 pregnant women between 1975 and 2021. The quality of the included studies showed that the majority of the studies were of intermediate to high quality [21].

Emphasis was placed on pollution with PM_2.5_ and PM_10_. Among the included studies, 33 examined the impact of PM_10_ [22,25,26,28,30,31,36,38,41,42,43,47,49,54,56,57,61,64,65,66,67,71,73,75,76,77,78,79,81,82,84,86,87] while 35 analyzed the impact of PM_2.5_ [23,24,25,26,27,28,30,31,32,33,34,35,36,39,40,41,43,44,46,50,51,52,53,54,55,57,58,59,60,63,66,67,71,74,78] and showed an association with SGA or TLBW. This was particularly noticeable in the case of PM_2.5_, for which the association with SGA was far more frequently seen than for PM_10_. Of the papers describing the influence of PM_10_, 55% showed an association between PM_10_ and SGA (Table 2 provides a detailed description of the included studies), while for PM_2.5_, this was as high as 74% (Table 3 provides a detailed description of the included studies). The average Odds Ratio (OR) of PM_10_ exposure influence on TLBW occurrence was 1.138 (minimal-maximal: 1.02–1.57) and 1.338 (minimal-maximal: 1.02–4.3) for PM_2.5_. Only seven papers performed analyses to estimate the influence of PM_10_ [22,25,43,57,64,65,66] and seven examined the influence of PM_2.5_ on SGA occurrence [23,27,33,43,44,58,74]. There were also two papers that showed a paradoxical protective effect of both PM_10_ (aOR 0.72) (95%CI: 0.56–0.92) and PM_2.5_ (aOR 0.86) (95%CI: 0.81–0.92) on TLBW [47,53].

A total of 16 studies showed an association between NO_x_ and the occurrence of SGA or TLBW [26,28,30,38,43,48,49,50,57,63,64,68,72,74,81,86]. This represents 66% of the papers about NO_x_ included in the review. Table 4 provides a detailed description of the included studies. The average OR of the influence of NO_x_ exposure on TLBW occurrence was 1.12 (min-max: 1.04–1.89). Only five papers performed analyses to estimate the influence of NO_x_ on SGA occurrence [43,49,50,72,74]. One study found a counterintuitive protective effect of exposure to NO_2_ substances during pregnancy with aOR 0.9 (95%CI: 0.93–0.95) [24].

A total of 12 studies showed an association between O_3_ and the occurrence of SGA or TLBW. Approximately 66% of the works selected for review showed a positive association between O_3_ and SGA or TLBW [26,28,36,37,43,47,53,58,64,73,74,79]. One study showed a protective effect of prenatal exposure to O_3_ [43]. The average OR of the influence of O_3_ exposure on TLBW occurrence was 1.173 (min-max: 1.02–1.48). Only two papers performed analyses to estimate the influence of O_3_ on SGA occurrence [43,64]. Interestingly, two studies reported that O_3_ exposure is associated with an increased incidence of macrosomia with OR 1.02 (95%CI: 1.017–1.03) [30,36]. Table 5 provides a detailed description of the included studies.

A total of 14 Studies examined SO_2_ [24,25,28,36,38,54,57,73,77,79,80,82,86,87] and 12 studies on CO [25,32,36,57,59,64,78,80,81,87,88,90] analyzed in the review showed a significant association with SGA and TLBW, with 87% of papers indicating an association for SO_2_ and 73% for CO, respectively. Table 6 provides a detailed description of the included studies. The average OR of the influence of SO_2_ exposure on TLBW occurrence was 1.29 (min-max: 1.03–1.81), and for CO, the OR was 1.23 (min-max: 1.01–1.49). Three studies estimated the influence on SGA occurrence [24,25,64]. All eight studies about VOCs showed the influence of exposure on the incidence of SGA or TLBW [29,32,38,45,83,85,86,89]. However, the influence of the VOC could not be compared as each study analyzed a different molecule.

Proximity to major roads was shown to be a risk factor for SGA and TLBW in four studies [63,69,70,74].

In the reviewed studies, air pollution measurements were based on data from research stations monitoring air quality in the area inhabited by the study cohorts. In all works, exposure assessment consisted of extracting pollution data from national or regional air quality databases. The assessment of individual pollution exposure was determined based on the residence location of a mother relative to the locations of the monitoring sites during a given time window using models such as Distributed Lag Models (DLMs), the General Additive Model (GAM), and the Land Use Regression Model (LUR). This process is called spatial-temporal exposure assessment and is one of the most popular methods used in air quality research. It involves using statistical models to determine the relationship between the level of air pollution and landscape characteristics and land use within a given area. This method can be used to estimate the level of air pollution based on geographic data, such as terrain maps, traffic flow, pollutant emission sources, and other variables. Geocoding the addresses of study participants is also a popular method used in air quality research. It involves assigning geographic coordinates to the addresses of study participants, enabling a comprehensive analysis of the relationship between the level of air pollution and geographical location.

The studies included in the systematic review presented here usually divided patients into two populations: those exposed to a specific air pollutant and those not exposed to that pollutant. The most common division was the quartile (Q) division, which was present in 54 studies [23,24,25,26,27,28,30,31,32,33,34,35,36,37,38,40,41,42,43,46,47,49,50,51,53,54,55,56,57,58,59,60,61,62,63,64,65,68,69,70,71,72,73,74,75,76,77,78,79,80,81,84,86,87]. The authors provided a cut-off point for those not exposed to the pollution, set at the I Q. In two studies, the quintile division by analogy as the exposed group establishing the I quintile was used [25,90]. Four papers divided the population into tertiles [48,82,83,85]. Three papers considered patients above the median concentration of air pollutants as the exposed group [66,67,88]. In addition, three papers used the World Health Organisation (WHO), European Union (EU), and United States of America Environmental Protection Agency (US EPA) air quality guidelines in their exposure criteria [22,39,44]. In yet another three papers, the authors arbitrarily set a cut-off level for the exposed and unexposed groups due to the lack of specific and unambiguous norms of concentration for the substances they analyzed [29,52,89].

Upon evaluation, the majority of the included studies demonstrated a moderate to high quality, as assessed using the Newcastle–Ottawa Scale [21]. All but 12 studies were adjusted for associated variables such as maternal age, BMI, pregnancy, ethnicity, and socioeconomic status. Those without aOR scored lower on the Ottawa–Newcastle scale [43,50,51,63,68,72,74,79,80,82,88,90]. The studies were based on retrospective and prospective cohorts compared with adjusted healthy pregnancies, producing high-quality results. Each study in the table has its Newcastle–Ottawa risk bias score (NOS) listed [21]. The detailed quality assessment of the included studies is presented in Supplementary Table S2.

## 4. Discussion

Most of the included studies showed an established direct association between ambient air pollution and the incidence of SGA or TLBW [22,23,24,25,26,27,28,29,30,31,32,33,34,35,36,37,38,39,40,41,42,43,44,45,46,47,48,49,50,51,53,54,55,57,58,59,60,61,63,64,65,66,67,68,72,73,74,76,77,78,79,80,81,82,83,84,85,86,87,88]. The pattern in the results above suggests that pollutants such as PM_2.5_, PM_10_, SO_2_, CO, and NO_2_ significantly impact low birth weight. Moreover, some studies indicated that a reduction in pollution concentration (NO_2_, PM_10_, etc.) is positively associated with increased birth weight [22,50,84]. Many studies consistently showed that there is a significantly increased risk of SGA for each 10 μg/m3 increase in PM_10_ and PM_2.5_ during pregnancy [26,27,33,34,51,72]. Most of the analyzed studies emphasize the harmful impact of these pollutants on fetal birth weight. On the other hand, a small proportion of the studies indicated a potential protective effect of certain air pollutants such as PM_2.5_, PM_10_, O_3_, and NO_x_ in reducing the incidence of SGA or TLBW [24,43,47,53]. Studies conducted by Huang et al. and Shang et al. established that O_3_ exposure was linked to increased term birth weight and a higher incidence of macrosomia [30,36].

### 4.1. PM_2.5_ and PM_10_ Exposure

There are four critical pollutants that the WHO considers crucial to human health: particulate matter, O_3_, NO_x_, and SO_2_. Despite the relatively large amount of epidemiological data on the impact of particulate matter, epidemiological data on gaseous pollutants are less abundant, especially regarding nitrogen compounds and sulfur dioxide.

PM_10_ and PM_2.5_ are atmospheric aerosols smaller than 10 and 2.5 micrometers in diameter, respectively. The toxicity of the pollutants is the result of many factors, including the location of deposition, which is different depending on particle size and reactivity [91]. The smaller the particles are, the more they sediment into the lower airways, which allows them to affect the alveolar–capillary barrier directly [13]. They trigger cytotoxicity, leading to a local and systemic inflammatory response (via cytokines and mediators) [92]. Similar results were shown in a meta-analysis conducted by Liu et al. [93].

PMs trigger pro-inflammatory signals through a Reactive Oxygen Species-dependent mechanism [94]. Oxidative stress, characterized by an imbalance between oxidants and antioxidants, can cause cell damage by oxidizing nucleic acids, proteins, and lipids, leading to cell death via apoptosis or necrosis [95]. There is much high-quality evidence in the literature from in vivo studies that chronic exposure to PM_2.5_ increases serum Interleucin 6 (IL-6), Tumor Necrosis Factor alpha (TNF-α), total cholesterol (TC), and Low-density lipoprotein C (LDL-C) levels, increases the expression of oxidative stress-related genes, causes progression of atherosclerosis, and leads to increased inflammation and redox levels in mice [95]. Increasing the antioxidant capacity of exposed cells has been shown to reduce the harmful effects of PM_2.5_ and PM_10_ [96].

It is speculated that PM_2.5_ and smaller particles (<0.1 um), called ultrafine particles (UFP), are able to reach other distant organs via the cardiovascular system [97]. The toxic effects of PM_2.5_ may be realized directly at the level of the placenta and the developing fetus, which in turn may trigger inflammation and oxidative stress, finally impeding trophoblast invasion, placental vascularisation via anti-angiogenic factors such as the sFlt-1 pathway, and placental dysfunction, a pivotal contributor to SGA [98,99,100]. This smaller size may explain the demonstrated significantly higher incidence of SGA for PM_2.5_.

In studies by Fernando Costa Nascimento et al. and Brown et al., a paradoxical protective effect of both PM_2.5_ and PM_10_ on TLBW was shown [47,53]. The possible reason for these negative associations may be the fact that high levels of exposure to air contamination throughout gestation led to miscarriage or stillbirth, which was not included as an outcome in those studies, thereby resulting in a selective survival bias for healthier fetuses.

### 4.2. O_3_ Exposure

O_3_ has the most evidence linking it to adverse health effects among gaseous pollutants. It is a pollutant formed by chemical reactions between nitrogen oxides (NO_x_) and VOCs in the presence of sunlight. In addition to being a highly reactive molecule capable of inducing oxidative stress, O_3_ has been shown to stimulate the synthesis of inflammatory cytokines by alveolar macrophages, such as IL-1ß, IL-6, IL-8, and TNF-α [101]. Studies conducted by Huang et al. and Shang et al. established that O_3_ exposure was linked to increased term birth weight and a higher incidence of macrosomia [30,36], and the study of Nobles et al. showed a protective effect of exposure to O_3_ [43]. This phenomenon might be explained by the observation by Beckerman et al. that O_3_ concentrations increased with proximity to the expressway, possibly due to O_3_ being scrubbed by NO to form NO_2_ [102]. The negative association with O_3_ may suggest a low level of exposure to TRAP, which may result in decreased SGA or TLBW occurrence.

### 4.3. Exposure to Traffic-Related Air Pollutants (TRAPs)

As almost every study shows, inconsistent results could result from exposure to multiple air pollutants. For example, in the study of Gan et al., while SO_2_ was found to have a significant impact on the prevalence of SGA, its exposure effects were reported in conjunction with other pollutants [28]. Evidence from car emissions studies emphasizes that the combined effect of air pollutants should be recognized as a primary risk factor for SGA [63,69,70,74]. While the composition of emissions may vary depending on differences in fuel type between gasoline and diesel vehicles [103], the emissions contain all of the pollutants evaluated in our study (PM_2.5_, PM_10_, O_3_, CO, NO_x_, SO_2_, VOCs, and more) [104,105]. Hence, the studies where only the influence of one pollutant was assessed neglect the combined, synergistic effect of other pollutants. It is also important to note that numerous factors potentially influence placental function and increase SGA risk. Only in some studies was the OR adjusted, and this should be considered when interpreting the findings from those studies.

### 4.4. NO_x_ Exposure

NO_x_ contributes to oxidative stress by generating reactive oxygen species that can overpower the placenta’s natural antioxidant barriers, causing cellular and molecular harm. NO_x_ exposure can cause inflammation in placental tissues, leading to functional damage and disrupting the exchange of nutrients and oxygen between the mother and fetus [106]. The inflammatory environment in the placenta can cause decreases in placental blood flow by constricting blood vessels and impairing endothelial function [107]. Furthermore, a discrepancy in the levels of NO and NO_2_ within blood vessels might lead to endothelial dysfunction, negatively impacting placental blood flow. The severity of these adverse outcomes depends on the level and duration of NO_x_ exposure. These alterations could lead to SGA and TLBW [26,28,30,38,43,48,49,50,57,63,64,68,72,74,81,86].

In the study by Mitku et al., the counterintuitive protective effect of exposure to NO_2_ was shown. The protective effect may result from simultaneous exposure to other environmental substances with a stronger protective impact, which conceals the negative effects of NO_2_ [24].

### 4.5. SO_2_ Exposure

There are few studies about the mechanism of SO_2_-induced changes in fetal weight [24,25,28,36,38,54,57,73,77,79,80,82,86,87]. It has been theorized that SO_2_ may cause changes in inflammatory factors in the blood, oxidative stress response, and deoxyribonucleic acid (DNA) methylation. Reactome pathway analysis showed that mainly NOTCH gene signalling was involved in genes associated with prenatal SO_2_ exposure [108].

### 4.6. CO Exposure

The fetotoxic effect of CO is associated with impaired cellular respiratory function. It irreversibly binds to the hemoproteins (cytochrome a-3 and myoglobin) that carry oxygen in the cell, leading to cellular respiration dysfunction. This results in mitochondrial degradation in CNS and heart cells, which require higher energy levels, cellular damage, and ultimately irreversible tissue damage. It also promotes the formation of oxygen-free radicals [109]. At the supracellular level, it prevents hemoglobin from delivering oxygen to tissues. The affinity of CO for hemoglobin is stronger in the fetus compared to children and adults. It is important to remember that fetal damage can occur even if the mother’s CO levels are not toxic [110], which could result in the appearance of SGA or TLBW [25,32,36,57,59,64,78,80,81,87,88,90].

### 4.7. VOC Exposure

Various potential processes have been proposed, including the impact of VOCs on developing fetuses, its influence on blood viscosity, and its effect on placental perfusion efficiency on the maternal side. Polycyclic aromatic hydrocarbons (PAHs) are believed to have a direct impact on fetal development and DNA transcription [29,111]. These air pollutants can impact maternal well-being by affecting the cardiovascular system and causing metabolic alterations. Consequently, there is a reduction in blood supply to the placenta, resulting in a higher occurrence of SGA [29,32,38,45,83,85,86,89]. Nevertheless, there was insufficient data to compare VOC substances in different populations, as each study analyzed a different molecule. Therefore, more studies are needed to compare these substances to other pollutants, such as PM_2.5_, PM_10_, SO_2_, O_3_, or NO_x_.

### 4.8. Exposure at a Particular Time of Pregnancy

Another important conclusion from the study is the association regarding exposure time. There appear to be specific windows during which the fetus is especially vulnerable or resistant to harmful substances, including air pollutants. Some studies have shown a positive association between SGA and exposure to harmful substances at any time during the pregnancy [25,26,30,37,76]. Other studies show a more significant influence in the first trimester, which is vital for organogenesis [26,34,40,57,63,64,72,76]. Nevertheless, it is believed that the influence in the first trimester has a binary effect on the pregnancy, either leading to a miscarriage or not leaving the pregnancy unaffected by any adverse consequences during pregnancy [26], as was shown by Bai et al. and Liu et al. in their meta-analyses assessing pregnancy outcomes including miscarriage [112,113]. The influence in the second and third trimesters may better reflect the influence of air pollutants on SGA as organogenesis is almost complete, and the fetus mainly grows during this period [25,31,33,37,43,49,59,64,72,76,81,87,88]. Compounds such as PM_10_ or PM_2.5_ can enter the bloodstream, accumulate in the fetal circulation, and cause oxidative stress. Chronic inflammation during pregnancy prevents the developing fetus from effectively utilizing nutrients to build reserves of adipose tissue, which is most intense in the third trimester.

Another possible cause for SGA after exposure to pollutants in the second and third trimesters could be individually and collaboratively mediated by increases in maternal blood pressure and hemoglobin levels caused by PM_2.5_, PM_10_, or CO. Hence, monitoring and controlling the mother’s blood pressure and hemoglobin levels during prenatal care may lower the risk of SGA through gestational exposure to PM_2.5_ [33]. An additional analysis of fetal growth restriction, especially its early and late variants, could explain the influence of air pollutants during the second and third trimesters of pregnancy. The discrepancies observed in the analyzed studies regarding the timing and effect on the incidence of SGA after pollution exposure might arise due to exposure to different pollutants, varying concentrations, or socioeconomic differences. These factors should be included in further prospective studies and evaluated using multivariate regression models.

### 4.9. Clinical Implementation and Further Research Directions

This study emphasizes the pressing concern of air pollution in a broader context, extending beyond just greenhouse gases responsible for global climate change. Air pollutants, including SO_2_, PM_10_, PM_2.5_, O_3_, and NO_x_ compounds, can negatively impact the maternal body and the developing fetus. Research has shown that being exposed to air pollution can alter placental DNA methylation, which may lead to disturbances in placental trophic function. This, in turn, can impede the delivery of nutrients to the developing fetus and obstruct the elimination of metabolic waste products from the fetal system [54].

Comparing all air pollutants, it seems that PM_2.5_ has the most influence on SGA and TLBW occurrence, as the OR of these pollutants seems to be most associated with the evaluated pregnancy complications. Nevertheless, further investigation of separate components of PM should be performed to evaluate the substances that affect the fetuses most. This knowledge is relevant as this compound could be eliminated from our ecosystem or minimized in global production, thus improving maternal and fetal outcomes.

The precise levels of heavy metals, a notable component of PM, have not been extensively examined in previous research. Prospective investigations are needed to measure these different components in particulate matter systematically and to comprehend their distinct impacts on air quality and potential perinatal complications, such as SGA or TLBW occurrence. This detailed method could result in a deeper comprehension of the risks linked to air pollution [114].

### 4.10. Strength and Limitations

To the best of our knowledge, this study is the latest and most comprehensive investigation of the effects of air pollution on the occurrence of SGA or TLBW infants. A particular strength of this review is the wide range of studies included and their number. The included research papers have evaluated the influence of exposure to different air pollutants on SGA or TLBW, with most being of moderate to high quality. The studies included in our analysis were mainly retrospective and did not account for specific molecular quantities in particulate matter, such as heavy metals. This exclusion is significant since typical pollution measurements often do not include such information. The lack of precise data highlights the necessity for a more detailed method of measuring pollution that can accurately quantify individual pollutant concentrations.

Nevertheless, the study has its limitations. One limitation is the absence of a cohort entirely unexposed to air pollution. Unfortunately, such a cohort is impossible to find because pollution-free environments are rare. Therefore, our study compared the effects of pollution between groups experiencing the highest exposure and those with the lowest measured exposure. Such division accurately reflects the living conditions among different populations across continents, considering similar national, geographic, cultural, and social backgrounds. Our outcomes might have been affected by the uneven distribution of the study and control groups, especially in terms of air quality and related factors. The different criteria and possible bias of the various studies may be significant weaknesses. The study group might live in regions with more industrial activity and air pollution; however, they also have better access to advanced healthcare facilities and higher levels of public health awareness. This difference in comparison to the control group, who may live in less polluted areas with restricted access to modern fetal monitoring, could unintentionally impact the reported occurrence of SGA and TLBW because of varying standards of medical care. However, these differences seem to be similar in the included studies.

Another limitation is the inability to monitor individual exposure precisely. The results of the included studies are based on estimating the level of air pollution based on the place of residence during pregnancy. The literature indicates that about one-quarter of pregnant women change their location during pregnancy [67]. One significant limitation was that the results were too heterogeneous to perform a meta-analysis. A notable oversight in several studies was that they failed to evaluate the confounding effect of maternal smoking, as smoke also contains several air pollutants that might influence SGA or TLBW rates. Finally, since it was effectively a combination of air pollutants that was assessed and its makeup changes based on location, assessing the influence of each pollutant proved challenging.

## 5. Conclusions

Air pollution is a real threat to the health of both the mother and the fetus. It is a global problem that is particularly pronounced in large urban areas. The ongoing industrialization and urbanization of society, especially in developing countries, will lead to the even greater exposure of pregnant women to air pollution, such as PM_2.5_, PM_10_, O_3_, NO_2_, CO, SO_2_, and other less-discovered pollutants. Patients in the high-risk group for SGA living in large cities and residing close to sources of pollution increase their risk of pregnancy complications, postpartum complications, and developmental issues. Relocating to less polluted environments can reduce the risk of SGA and may benefit the mother and the fetus. The nature, magnitude, and timing of pollutant exposure influence pregnancy outcomes. Understanding the detailed mechanisms of the effect of air pollution during pregnancy and identifying the most vulnerable windows during pregnancy require further research. Clarifying the exact exposure–time association will improve SGA prevention, especially in high-risk pregnancies. Using standardized exposure criteria and methods for individual assessments of exposure to air pollutants will improve our understanding of this pressing issue, one of the biggest challenges of our times.

There is a strong need to objectify individual exposure to pollutants, which can be achieved by prospective cohort studies and measurement of personal exposure or blood biomarkers. Systematizing exposures will allow better characterization of the association between air pollution and adverse birth outcomes.

However, it is essential to note that finding a place on Earth unaffected by air pollution is almost impossible. Thus, humans must adapt to escalating environmental pollution or undertake significant steps to stop the pollution of the Earth.

## Figures and Tables

**Table 1:** Search strategy.

(pregnant OR pregnancy OR fetus OR foetus OR foetal OR fetal) AND (“air pollution” OR “air pollutants” OR PM_10_ OR PM_2.5_ OR ozone OR CO OR NO_2_ OR NO_x_ OR SO_2_ OR VOC OR “particulate matter” OR particulates OR “ground ozone” OR “carbon monoxide” OR “volatile organic compounds” OR “nitrogen dioxide” OR “sulfur dioxide” OR “sulphur dioxide”) AND (“birth weight” OR “hypotrophy” OR “small for gestational age” OR SGA OR “intrauterine growth restriction” OR “fetal growth restriction” OR “term low birth weight” OR “low birth weight” OR TLBW OR LBW AND (Infant, Low Birth Weight [MeSH]))

**Table 2:** Characteristics of the included studies on the influence of Particulate Matter ≤ 10 μm (PM 10).

Study	Time and Place of Exposure Type of Pollutant	Character of the Study and Number of Included Patients			Outcomes
			Study Group	Control Group	
Canto et al. (2023) [22]	2009–2010 Spain Pollutant: PM_10_	Retrospective study n = 288,229	Exposure cut-offs of PM_10_ 15–19 μg/m^3^ (n = 50,967) 20–24 μg/m^3^ (n = 123,601) 25–29 μg/m^3^ (n = 90,474) 30–34 μg/m^3^ (n = 15,388) 35–39 μg/m^3^ (n = 2276) 40–44 μg/m^3^ (n = 323) 45–49 μg/m^3^ (n = 100) 50–54 μg/m^3^ (n = 54) 55–59 μg/m^3^ (n = 37) 60–64 μg/m^3^ (n = 1)	Exposure cut-off of PM_10_: ≤15 μg/m^3^ (n = 5008) and ≤40 μg/m^3^ (n = 287,714)	PM_10_ exposure is related to SGA (adjusted odds ratio (aOR) 1.05, 95 % confidence interval (CI): 1.0–1.09). Reduction of 10 μg/m^3^ of PM_10_ was associated with an increase of 22 g, 95 % CI: 17.2–28.0). 15 % and 50 % reduction of PM_10_ exposure reduces risk of term low birth weight (TLBW) and small for gestational age (SGA) occurrence.
Zhou et al. (2023) [26]	2015–2020 Chongqing, China Pollutants: PM_2.5_, PM_10_, NO_2_, CO and O_3_	Retrospective study n = 572,106	Number of exposed were not specified. Exposure cut-off of PM_10_ in II–IV Quartile (Q): 59.1–121.5 μg/m^3^	Number of nonexposed were not specified. Exposure cut-off of PM_10_ in I Q: 28.8–59.1 μg/m^3^	10 μg/m^3^ increase in PM_10_ exposure is related to VTLBW occurrence (RR 1.13, 95%CI: 1.06–1.21).
Gan et al. (2022) [28]	2017–2018 Guangzhou, China Pollutants: PM_2.5_, NO_2_, SO_2_, O_3_, and PM_10_	Prospective study n = 916	Number of exposed were not specified. Exposure cut-off of PM_10_ in II–IV Q: Cut-off point of exposure not specified in study.	Number of nonexposed were not specified. Exposure cut-off of PM_10_ in I Q: Cut-off point of exposure not specified in study.	TLBW is associated with maternal exposure to: SO_2_ and PM_10_ (OR 1.23, 95%CI: 1.03–1.46)
Huang et al. (2022) [30]	2015–2016 Wen Zhou, China Pollutants: PM_2.5_, PM_10_, SO_2_, NO_2_, and O_3_	Retrospective study n = 213,959	Number of exposed were not specified. Exposure cut-off of PM_10_ in II–IV Q: 66.2–86.0 μg/m^3^	Number of nonexposed were not specified. Exposure cut-off of PM_10_ in I Q: <66.2 μg/m^3^	TLBW is associated with maternal exposure to PM_10_ (aOR 1.14, 95%CI: 1.06–1.23) during the entire pregnancy. The significant influence was shown especially in the 2nd trimester.
Rodríguez-Fernández et al. (2022) [31]	2014–2016 Chile Pollutants: PM_2.5_ and PM_10_	Cross sectional study n = 595,369	Number of exposed were not specified. Exposure cut-off of PM_10_ in II–IV Q: Cut-off point of exposure not specified in study.	Number of nonexposed were not specified. Exposure cut-off of PM_10_ in I Q: Cut-off point of exposure not specified in study.	Second trimester exposure of PM_10_ (aOR 1.14, 95%CI: 1.11–1.18) is associated with an increased the risk of TLBW
Shang et al. (2021) [36]	2015–2018 Xi’an city of Shaanxi, China Pollutants: high level of air quality index (AQI), PM_2.5_, PM_10_, SO_2_, CO, O_3_, NO_2_	Retrospective study n = 321,521	Number of exposed were not specified. Exposure cut-off of PM_10_ in II–IV Q: >73.9 μg/m^3^.	Number of nonexposed were not specified. Exposure cut-off of PM_10_ in I Q: <73.9 μg/m^3^.	TLBW is associated with maternal exposure to PM_10_ (OR 1.02, 95%CI: 1.009–1.03)
Enders et al. (2019) [41]	2002–2013 California, USA Pollutants: PM_10_ and PM_2.5_	Retrospective study n = 2,719,596	Number of exposed were not specified. Exposure cut-off of PM_10_ in II–IV Q: II Q of PM_10_ (11.4–14.3 μg/m^3^) III Q of PM_10_ (14.3–18.5 μg/m^3^) IV Q of PM_10_ (>18.5 μg/m^3^)	Number of nonexposed were not specified. Exposure cut-off of PM_10_ in I Q: <11.4 μg/m^3^.	TLBW is associated with maternal exposure to PM_2.5-10_ in II Q (aOR 1.00, 95%CI: 0.98–1.03), III Q (aOR 1.03, 95%CI: 1.00–1.06).
Kim et al. (2019) [42]	2010–2013 Korea Pollutant: PM_10_	Retrospective study n = 1,742,183	Number of exposed were not specified. Exposure cut-off of PM_10_ in IV Q: >70 μg/m^3^)	Number of nonexposed were not specified. Exposure cut-off of PM_10_ in I–II Q: <70 μg/m^3^.	The rate of low birth weight in term infants increased when women were exposed to > 70 µg/m^3^ PM_10_ (aOR 1.060, 95%CI: 0.953–1.178)
Nobles et al. (2019) [43]	2002–2010 20 hospitals in USA Pollutants: SO_2_, O_3_, NO_x_, NO_2_, CO, PM_10_ and PM_2.5_	Retrospective study n = 109,126 births	Number of exposed were not specified. Exposure with SO_2_, O_3_, NO_x_, NO_2_, CO, PM_10_, PM_2.5_ from II–IV Q Quartiles of exposure cut-offs not specified in study.	Number of nonexposed were not specified. Exposure with SO_2_, O_3_, NO_x_, NO_2_, CO, PM_10_, PM_2.5_ in I Q Quartiles of exposure cut-offs not specified in study.	Risk of SGA increases in the third trimester every 10th percentile per interquartile increase in exposure of PM_10_ (RR 1.03, 95%CI: 1.00–1.06).
Costa Nascimento et al. (2017) [47]	2012–2013 São José do Rio Preto, Brazil Pollutants: NO_2_, PM_10_ and O_3_	Retrospective longitudinal study n = 8948	Number of exposed were not specified. Exposure cut-off of PM_10_ in II–IV Q: 33.47–65.66 μg/m^3^.	Number of nonexposed were not specified. Exposure cut-off of PM_10_ in I Q: <33.47 μg/m^3^.	Exposure to PM_10_ had a paradoxical protective effect (aOR 0.72, 95%CI: 0.56–0.92) on TLBW occurrence.
Habermann and Gouveia (2014) [56]	2006 Sao Paulo, Brazil Pollutant: PM_10_	Retrospective study n = 11,586	8613 pregnant women exposed with traffic related air pollution of PM_10_ from second to fourth quartile. Exposure cut-off of PM_10_ in: II Q (35.3–37.0 μg/m^3^) III Q (37.0–40.4 μg/m^3^) IV Q (40.4–108.2 μg/m^3^).	2952 pregnant women exposed with traffic related PM_10_ from first quartile. Exposure cut-off of PM_10_ in I Q: < 35.3 μg/m^3^.	PM_10_ exposure measured with LUR-PM_10_ is not related to TLBW.
Hannam et al. (2014) [57]	2004–2008 Northwest England, UK Pollutants: NO_x_, NO_2_, CO, PM_2.5_ and PM_10_	Retrospective study n = 203,562	Number of exposed were not specified. Exposure cut-off of PM_10_ in II–IV Q: 46.3 ≥ 69.8 μg/m^3^.	Number of nonexposed were not specified. Exposure cut-off of PM_10_ in I Q: 18.3–35.4 μg/m^3^.	NO_x_, NO_2_, CO, PM_2.5_, PM_10_ is related with increased risk of SGA infant. Small statistically significant association was observed for PM_10_ and SGA, particularly with exposure in the first and third trimesters. Similar effects on SGA were also found for NO_2_, PM_2.5_, and CO in later pregnancy, but no overall increased risk was observed. NO_2_ (aOR 1.66, 95%CI: 1.47–1.87), PM_10_ (aOR 1.57, 95%CI: 1.43–1.71).
Candela et al. (2013) [61]	2003–2010 Emilia-romagna region, UK Pollutants: PM_10_	Retrospective study n = 21,517	16,731 pregnant women exposed with PM_10_ and NO_x_ form second quintile to fifth quintile. Exposure cut-off of PM_10_ in: II Q: 0.08–0.2 ng/m^3^ III Q: 0.2–0.3 ng/m^3^ IV Q: >0.3–0.8 ng/m^3^.	4433 pregnant women exposed with PM_10_ in first quintile. Exposure cut-off of PM_10_ in I Q: <0.07 ng/m^3^.	No associations were observed between PM_10_ exposure and SGA or TLBW occurrence.
Le et al. (2012) [64]	1990–2001 Detroit, Michigan, USA Pollutants: CO, NO_2_, PM_10_ and O_3_	Retrospective study n = 164,905	Number of exposed were not specified. Exposure cut-off of PM_10_ in II–IV Q: >35 μg/m^3^.	Number of nonexposed were not specified. Exposure cut-off of PM_10_ in I Q: <35 μg/m^3^.	SGA was associated with PM_10_ (aOR 1.22, 95%CI: 1.03–1.46). Third trimester top-quartile PM_10_ exposure (>35.8 μg m^3^) gave the highest risk of a term SGA birth (aOR 1.22, 95%CI: 1.04–1.44)
van den Hooven et al. (2012) [65]	2001–2005 Rotterdam, Netherlands Pollutants: PM_10_ and NO_2_	Prospective study n = 7772	6928 pregnant women exposed with PM_10_, NO_2_ in II–IV Q. Exposure cut-off of PM_10_ in II–IV Q: 27.8–40.9 μg/m^3^.	844 pregnant women exposed with PM_10_, NO_2_ in I Q. Exposure cut-off of PM_10_ in I Q: <27.8 μg/m^3^	III Q of PM_10_ exposure is related with SGA (aOR 1.38, 95%CI: 1.00–1.90).
Salihu et al. (2012) [66]	2000–2007 Tampa, Florida, USA Pollutants: PM_2.5_ and PM_10_	Retrospective study n = 12,356	8791 pregnant women exposed with PM_2.5_, PM_10_ above the median. Exposure above the median: >25.04 μg/m^3^ PM_10_	3565 pregnant women exposed with PM_2.5_, PM_10_ below the median. Exposure below the median: <25.04 μg/m^3^ PM_10_	Women exposed to air particulate pollutants were at elevated risk for TLBW (aOR 1.24, 95%CI: 1.07–1.43), VLBW (aOR 1.58, 95%CI: 1.09–2.29) SGA was related to PM_10_ exposure (aOR 1.14, 95%CI: 1.03–1.27).
Salihu et al. (2012) [67]	2000–2007 Tampa, Florida, USA Pollutants: PM_2.5_ and PM_10_	Retrospective study n = 103,961	24,090 pregnant women exposed with PM_2.5_, PM_10_ above the median. Exposure above the median: >24.35 μg/m^3^ PM_10_	79,871 pregnant women exposed with PM_2.5_, PM_10_ below the median. Exposure below the median: <24.35 μg/m^3^ PM_10_	Exposed women had increased odds for low birth weight and very low birth weight, with the greatest risk being for very low birth weight (aOR 1.27, 95%CI 1.08–1.49). TLBW was related to PM_10_ exposure (aOR 1.13, 95%CI: 1.07–1.19).
Madsen et al. (2010) [71]	1999–2002 Oslo, Norway Pollutants: NO_2_, PM_10_ and PM_2.5_	Retrospective study n = 25,229	18,921 pregnant women exposed with NO_2_, PM_10_, PM_2.5_ II–IV Q. Exposure cut-off of PM_10_ in II–IV Q PM_10_: >10.8 μg/m^3^.	6308 pregnant women exposed with NO_2_, PM_10_, PM_2.5_ in I Q. Exposure cut-off of PM_10_ in I Q: <10.7 μg/m^3^.	No associations were observed between NO_2_, PM_10_ exposure and SGA or TLBW occurrence.
Hansen et al. (2007) [75]	2000–2003 Brisbane, Australia Pollutants: PM_10_, NO_2_ and O_3_	Retrospective study n = 26,617	Number of exposed were not specified. Exposure cut-off of PM_10_ in II–IV Q: 14.6–171.7 μg/m^3^.	Number of nonexposed were not specified. Exposure cut-off of PM_10_ in I Q: <14.6 μg/m^3^.	No associations were observed between PM_10_, NO_2_, or O_3_ exposure and SGA or TLBW occurrence.
Kim et al. (2007) [76]	2001–2004 Seul, Korea Pollutant: PM_10_	Multicenter prospective study n = 1514	Number of exposed were not specified. Exposure with PM_10_ from II to IV Q. Cut-off points of exposure not specified in study.	Number of nonexposed were not specified. Exposure with PM_10_ in I Q. Cut-off points of exposure not specified in study.	IUGR was affected by the first trimester’s PM_10_ exposure. TLBW was affected by the PM_10_ level during the whole pregnancy. TLBW was affected by a 10 g/m^3^ increase in the average ambient PM_10_ concentration during the first (aOR 1.1, 95%CI: 1.0–1.2), second (aOR 1.1, 95%CI: 0.9–1.2), and third (aOR 1.1, 95%CI: 1.0–1.2) trimesters.
Dugandzic et al. (2006) [77]	1988–2000 Nova Scotia Atlee, Canada Pollutants: PM_10_, SO_2_ and O_3_	Retrospective study n = 74,284	Number of exposed were not specified. Exposure cut-off of PM_10_ in II–IV Q: 14–53 µg/m^3^.	Number of nonexposed were not specified. Exposure cut-off of PM_10_ in I Q PM_10_: < 14 µg/m^3^.	SO_2_ exposure during the I trimester is related with TLBW (RR 1.36, 95%CI: 1.04–1.78) PM_10_ exposure during the I trimester is related with TLBW (RR 1.33, 95%CI: 1.02–1.74).
Lin et al. (2004) [79]	1995–1997 Taipei and Kaohsiung, Taiwan Pollutants: SO_2_, PM_10_, CO, O_3_ and NO_2_	Retrospective study n = 31,530 (Kaohsiung) n = 60,758 (Taipei)	31,530 pregnant women from Kaohsiung exposed with mean concentration of: PM_10_ (65.8–83.6 μg/m^3^)	60,758 pregnant women from Taipei exposed with mean concentration of: PM_10_ (46.4–51.9 μg/m^3^).	Higher exposure of SO_2_, PM_10_, CO, O_3_, and NO_2_ in Kaohsiung leads to 13% higher TLBW occurrence than lower exposure in Taipei (OR 1.13, 95%CI: 1.03–1.24).
Lin et al. (2004) [80]	1995–1997 Taipei and Kaohsiung, Taiwan Pollutants: SO_2_, PM_10_, CO, O_3_ and NO_2_	Retrospective study n = 92,288	Number of exposed were not specified. Exposure cut-off of PM_10_ in II–IV Q PM_10_: >46.4 μg/m^3^	Number of nonexposed were not specified. Exposure cut-off of PM_10_ in I Q: <46.4 μg/m^3^.	No associations were observed between PM_10_ exposure and TLBW occurrence.
Lee et al. (2003) [81]	1996–1998 Seoul, Korea Pollutants: CO, PM_10_, SO_2_ and NO_2_	Retrospective study n = 388,105	Number of exposed were not specified. Exposure cut-off of PM_10_ in II–IV Q PM_10_: 47.4–236.9 μg/m^3^.	Number of nonexposed were not specified. Exposure cut-off of PM_10_ in I Q PM_10_: 18.4–47.4 μg/m^3^.	Second-trimester PM_10_ exposure increased the risk for TLBW (aOR 1.04, 95%CI: 1.00–1.08).
Chen et al. (2002) [84]	1991–1999 Nevada State, USA Pollutants: PM_10_, CO and O_3_	Retrospective study n = 39,338	32,676 pregnant women exposed with PM_10_ at the third trimester (>19.72 µg/m^3^).	3629 pregnant women with low exposure to PM_10_ at the third trimester (<19.72 µg/m^3^).	A 10 µg/m^3^ increase in PM_10_ level in the third trimester can be associated with a birth weight reduction of 11 g (95%CI: 2.3–19.8 g)
Lin et al. (2001) [86]	1993–1996 Lin-Yuan and Taicei, Taiwan Pollutants: SO_2_, NO_2_, PM_10_, SO_4_^2−^, NH_4_^+^ and NO_3_^−^	Retrospective study n = 2545	1677 pregnant women from Lin-Yuan municipality. Exposure cut-off of PM_10_ in II–IV Q: 85.9 ± 1.7 μg/m^3^.	868 pregnant women from Taicei municipality. Exposure cut-off of PM_10_ in I Q PM_10_: 59.2 ± 1.4 μg/m^3^.	Higher exposure of SO_2_, NO_2_, PM_10_, SO_4_^2−^, and NO_3_^−^ in a petrochemical municipality in Lin-Yuan leads to 3.22% TLBW occurrence in comparison to lower exposure in a control municipality Taicei which led to 1.84% TLBW occurrence.

**Table 3:** Characteristics of included studies about the influence of Particulate. Matter ≤ 2.5 μm (PM 2.5).

Study	Time and Place of Exposure Type of Pollutant	Character of the Study and Number of Included Patients			Outcomes
			Study Group	Control Group	
Chen et al. (2023) [23]	2014–2018 8 provinces in China Pollutant: PM_2.5_	Prospective study n = 179,761	Number of exposed were not specified. Exposure with PM_2.5_ in II–IV Q. Cut-off point of exposure not specified in study.	Number of nonexposed were not specified. Exposure with PM_2.5_ in I Q. Cut-off point of exposure not specified in study.	PM_2.5_ exposure is related with SGA occurrence (aOR 1.02, 95 % CI: 1.01–1.04)
Mitku et al. (2023) [24]	2013–2017 Durban, South Africa Pollutants: PM_2.5_, SO_2_, NO_x_ (NO and NO_2_)	Retrospective study n = 656 from low socioeconomic neighbourhoods	Number of exposed were not specified. Exposure cut-off of PM_2.5_ in II–IV Q: Cut-off point of exposure not specified in study.	Number of nonexposed were not specified. Exposure cut-off of PM_2.5_ in I Q: Cut-off point of exposure not specified in study.	Increased SGA occurrence risk is associated with exposure to PM_2.5_ (aOR 1.2, 95%CI: 1.21–1.28) and SO_2_ (aOR 1.1, 95%CI: 1.01–1.13).
Zhou et al. (2023) [26]	2015–2020 Chongqing, China Pollutants: PM_2.5_, PM_10_, NO_2_, CO and O_3_	Retrospective study n = 572,106	Number of exposed were not specified. Exposure cut-off of PM_2.5_ in II–IV Q PM_2.5_: 34.4–83.7 μg/m^3^.	Number of nonexposed were not specified. Exposure cut-off of PM_2.5_ in I Q: 17.8–34.4 μg/m^3^.	10 μg/m^3^ increase in PM_2.5_ exposure is related to very low birth weight (VLBW) occurrence (relative risk (RR) 1.1, 95%CI: 1.01–1.2).
Ahmad et al. (2022) [27]	2004–2015 Israel Pollutant: PM_2.5_	Retrospective study n = 381,265	Number of exposed were not specified. Exposure cut-off of PM_2.5_ in II–IV Q: Cut-off point of exposure not specified in study.	Number of nonexposed were not specified. Exposure cut-off of PM_2.5_ in I Q: Cut-off point of exposure not specified in study.	10 μg/m^3^ increase in PM_2.5_ led to increased risk of TLBW (OR 1.25, 95%CI: 1.09–1.43) and SGA (OR 1.15, 95%CI: 1.06–1.26).
Gan et al. (2022) [28]	2017–2018 Guangzhou, China Pollutants: PM_2.5_, NO_2_, SO_2_, O_3_, and PM_10_	Prospective study n = 916	Number of exposed were not specified. Exposure cut-off of PM_2.5_ in II–IV Q: Cut-off point of exposure not specified in study.	Number of nonexposed were not specified. Exposure cut-off of PM_2.5_ in I Q: Cut-off point of exposure not specified in study.	TLBW is associated with maternal exposure to SO_2_ and PM_2.5_ (OR 1.28, 95%CI: 1.07–1.52).
Huang et al. (2022) [30]	2015–2016 Wen Zhou, China Pollutants: PM_2.5_, PM_10_, SO_2_, NO_2_, and O_3_	Retrospective study n = 213,959	Number of exposed were not specified. Exposure cut-off of PM_2.5_ in II–IV Q: 39.1–52.7 μg/m^3^.	Number of nonexposed were not specified. Exposure cut-off of PM_2.5_ in I Q: <39.1 μg/m^3^.	TLBW is associated with maternal exposure to PM_2.5_ (aOR 1.12, 95%CI: 1.02–1.24) during the entire pregnancy. A significant influence was shown, especially in the 2nd trimester.
Rodríguez-Fernández et al. (2022) [31]	2014–2016 Chile Pollutants: PM_2.5_ and PM_10_	Cross sectional study n = 595,369	Number of exposed were not specified. Exposure cut-off of PM_2.5_ in II–IV Q: Cut-off point of exposure not specified in study.	Number of nonexposed were not specified. Exposure cut-off of PM_2.5_ in I Q: Cut-off point of exposure not specified in study.	Second trimester exposure to PM_2.5_ (aOR 1.03, 95%CI: 1.004–1.06) is associated with an increased the risk of TLBW.
Shen et al. (2022) [32]	2015–2016 24 provinces in China Pollutants: PM_2.5_, CO, NH_4_^+^ (ammonium), SO_4_^2−^ (sulphate)	Retrospective study n = 70,206	Number of exposed were not specified. Exposure cut-off of PM_2.5_ in II–IV Q PM_2.5_: 41–110 μg/m^3^.	Number of nonexposed were not specified. Exposure cut-off of PM_2.5_ in I Q: <41 μg/m^3^.	PM_2.5_ exposure during pregnancy is associated with 16%, 95%CI: 3–30% higher risk of SGA.
Zhu et al. (2022) [33]	2014–2018 China Pollutant: PM_2.5_	Prospective study n = 117,162	Number of exposed were not specified. Exposure cut-off of PM_2.5_ in II–IV Q: >28 μg/m^3^.	Number of nonexposed were not specified. Exposure cut-off of PM_2.5_ in I Q: <28 μg/m^3^.	10 μg/m^3^ increase in PM_2.5_ exposure is correlated with increased SGA occurrence in the second trimester (OR 1.023, 95%CI: 1.008–1.037) and during the whole pregnancy (OR 1.025, 95%CI: 1.002–1.048)
Chen et al. (2022) [34]	2014–2016 most air-polluted cities in China Pollutant: PM_2.5_	Retrospective study n = 10,916	Number of exposed were not specified. Exposure cut-off of PM_2.5_ in II–IV Q: Cut-off point of exposure not specified in study.	Number of nonexposed were not specified. Exposure cut-off of PM_2.5_ in I Q: Cut-off point of exposure not specified in study.	10 μg/m^3^ increase in PM_2.5_ positively correlates to SGA occurrence in preconceptional time and in the first trimester. The strongest correlation is in the 5th week before conception (HR 1.06, 95%CI: 1.03–1.09).
Chen et al. (2021) [35]	1993–2005 UK Pollutant: PM_2.5_	Retrospective study n = 12,020	Number of exposed were not specified. Exposure cut-off of PM_2.5_ in II–IV Q: Cut-off point of exposure not specified in study.	Number of nonexposed were not specified. Exposure cut-off of PM_2.5_ in I Q: Cut-off point of exposure not specified in study.	PM_2.5_ exposure increased TLBW occurrence by 40% (OR 1.40, 95%CI: 1.12–1.75) and SGA occurrence by 18% (OR 1.18, 95%CI: 1.05–1.32)
Shang et al. (2021) [36]	2015–2018 Xi’an city of Shaanxi, China, Pollutants: high level of air quality index (AQI), PM_2.5_, PM_10_, SO_2_, CO, O_3_, NO_2_	Retrospective study n = 321,521	Number of exposed were not specified. Exposure cut-off of PM_2.5_ in II–IV Q PM_2.5_: >33.4 μg/m^3^.	Number of nonexposed were not specified. Exposure cut-off of PM_2.5_ in I Q: <33.4 μg/m^3^.	TLBW is associated with maternal exposure to PM_2.5_ (OR 1.02, 95%CI: 1.006–1.03).
Wojtyla et al. (2020) [39]	2016–2017 Poland Pollutant: PM_2.5_	Retrospective study n = 1095	634 pregnant women exposed with PM_2.5_ cut-off > 25 μg/m^3^.	432 pregnant women exposed with PM_2.5_ cut-off < 25 μg/m^3^.	Exposure to PM_2.5_ is related to SGA. It is 4 times more likely to lead to TLBW (aOR 4.3, 95%CI: 1.5–2.3)
Tapia et al. (2020) [40]	2012–2016 Lima, Peru Pollutant: PM_2.5_	Retrospective study n = 123,034	Number of exposed were not specified. Exposure cut-off of PM_2.5_ in II–IV Q: 16.84–41.6 μg/m^3^.	Number of nonexposed were not specified. Exposure cut-off of PM_2.5_ in I Q: 12.7–16.83 μg/m^3^.	SGA was associated with exposure to PM_2.5_ exposure overall (aOR 1.04, 95%CI: 1.01–1.08) and in the first (aOR 1.07, 95%CI: 1.03–1.10) and third trimesters (aOR 1.04, 95%CI: 1.00–1.07).
Enders et al. (2019) [41]	2002–2013 California, USAPollutants: PM_10_ and PM_2.5_	Retrospective study n = 2,719,596	Number of exposed were not specified. Exposure cut-off of PM_2.5_ in: II Q PM_2.5_ (10.2–12.6 μg/m^3^) III Q PM_2.5_ (12.6–16.1 μg/m^3^) IV Q PM_2.5_ (>16.1 μg/m^3^)	Number of nonexposed were not specified. Exposure cut-off of PM_2.5_ in I Q: <10.2 μg/m^3^.	TLBW is associated with maternal exposure to PM_2.5-10_ in II Q (aOR 1.00, 95%CI: 0.98–1.03) and III Q (aOR 1.03, 95%CI: 1.00–1.06). PM_2.5_ exposure correlates with TLBW in IV Q (aOR 1.04, 95%CI: 1.01–1.07).
Nobles et al. (2019) [43]	2002–2010 20 hospitals in USA Pollutants: SO_2_, O_3_, NO_x_, NO_2_, CO, PM_10_, PM_2.5_	Retrospective study n = 109,126 births	Number of exposed were not specified. Exposure cut-off of PM_2.5_ in II–IV Q: Cut-off point of exposure not specified in study.	Number of nonexposed were not specified. Exposure cut-off of PM_2.5_ in I Q: Cut-off point of exposure not specified in study.	Risk of SGA increases in the third trimester every 10th percentile per interquartile increase in exposure of PM_2.5_ (RR 1.02, 95%CI 1.00, 1.05).
Percy et al. (2019) [44]	2007–2010 Ohio, USA Pollutant: PM_2.5_	Retrospective study n = 224,921	181,665 pregnant women exposed with ≥15 μg/m^3^ PM_2.5_	43,256 pregnant women exposed with <15 μg/m^3^ PM_2.5_	III trimester exposure of PM_2.5_ increases SGA occurrence (aOR 1.09, 95%CI: 1.02–1.17)
Wu (2018) [46]	2013–2016 Jinan, China Pollutants: PM_2.5_, NO_2_, SO_2_	Retrospective study n = 43,855	Number of exposed were not specified. Exposure cut-off of PM_2.5_ in II–IV Q: 80.5–119.3 μg/m^3^.	Number of nonexposed were not specified. Exposure cut-off of PM_2.5_ in I Q: <80.4 μg/m^3^.	PM_2.5_ was positively associated with TLBW in II Q (aOR 1.77, 95%CI: 1.09–2.88), III Q (aOR 1.77, 95%CI: 1.03–3.04), and IV Q (aOR 1.92, 95%CI: 1.04–3.55)
Stieb et al. (2016) [50]	1999–2008 Canada Pollutants: PM_2.5_	Retrospective study n = 2,965,440	Number of exposed were not specified. Exposure cut-off of PM_2.5_ II–IV Q: Cut-off point of exposure not specified in study.	Number of nonexposed were not specified. Exposure cut-off of PM_2.5_ in I Q: Cut-off point of exposure not specified in study.	10 μg/m^3^ increase in PM_2.5_ exposure is related to 4% increase in SGA (OR 1.04, 95%CI: 1.01–1.07)
Lavigne et al. (2016) [52]	2005–2012 Ontario, Canada Pollutants: PM_2.5_, NO_2_, and O_3_	Retrospective study n = 818,400	Number of exposed were not specified. Exposure cut-offs of >5 percentile of PM_2.5_ (>6 μg/m^3^).	Number of nonexposed were not specified. Exposure cut-offs of ≤5 percentile of PM_2.5_ (≤6 μg/m^3^).	No associations were observed between PM_2.5_, NO_2_, or O_3_ exposure and SGA or TLBW occurrence.
Brown et al. (2015) [53]	2001–2006 New York, USAPollutants: O_3_ and PM_2.5_	Retrospective study n = 480,430	Number of nonexposed were not specified. Exposure cut-off of PM_2.5_ in II–IV Q: 9.75–18.07 μg/m^3^.	Number of nonexposed were not specified. Exposure cut-off of PM_2.5_ in I Q: 3.66–9.49 μg/m^3^.	There was paradoxical effect of decreased SGA occurrence after exposure to II Q PM_2.5_ (aOR 0.87, 95%CI: 0.79–0.96), and to the III Q of O_3_ (aOR 0.86, 95%CI: 0.81–0.92).
Twum et al. (2015) [55]	2004 9 counties of Georgia, USA Pollutant: PM_2.5_	Retrospective study n = 48,172	36,129 pregnant women exposed with PM_2.5_. Exposure cut-off of PM_2.5_ in II–IV Q: Cut-off point of exposure not specified in study.	12,043 pregnant women exposed with PM_2.5_. Exposure cut-off of PM_2.5_ in I Q: Cut-off point of exposure not specified in study.	75–95th percentile exposure of PM_2.5_ was related to TLBW (aOR 1.36, 95%CI: 1.03–1.79)
Hannam et al. (2014) [57]	2004–2008 Northwest England, UK Pollutants: NO_x_, NO_2_, CO, PM_2.5_, PM_10_	Retrospective study n = 203,562	Number of exposed were not specified. Exposure cut-off of PM_2.5_ in II–IV Q: 24.3 ≥ 41.0 μg/m^3^.	Number of nonexposed were not specified. Exposure cut-off of PM_2.5_ in I Q: 10.3–19.7 μg/m^3^.	NO_x_, NO_2_, CO, PM_2.5_, PM_10_ is related with increased risk of SGA infant. Small statistically significant association was observed for PM_10_ and SGA, particularly with exposure in the first and third trimesters. Similar effects on SGA were also found for NO_2_, PM_2.5_, and CO in later pregnancy, but no overall increased risk was observed.
Vinikoor-Imler et al. (2014) [58]	2003–2005 North Carolina, USA Pollutants: PM_2.5_ and O_3_	Retrospective study n = 312,638	Number of exposed were not specified. Exposure cut-off of PM_2.5_ in II–IV Q: Cut-off point of exposure not specified in study.	Number of nonexposed were not specified. Exposure cut-off of PM_2.5_ in I Q: Cut-off point of exposure not specified in study.	No associations were observed between PM_2.5_ exposure and SGA occurrence.
da Silva et al. (2014) [59]	2004–2005 Mato Grosso, Brazil Pollutants: PM_2.5_ and CO	Retrospective study n = 6642	Number of exposed were not specified. Exposure cut-off of PM_2.5_ in II–IV Q: Cut-off point of exposure not specified in study.	Number of nonexposed were not specified. Exposure cut-off of PM_2.5_ in I Q: Cut-off point of exposure not specified in study.	Second trimester exposure (IV Q) to PM_2.5_ (aOR 1.51, 95%CI: 1.04–2.17) is related to increased risk of TLBW.
Hyder et al. (2014) [60]	2000–2006 Massachusetts, USA Pollutants: PM_2.5_	Retrospective study n = 834,332	Number of exposed were not specified. Exposure cut-off of PM_2.5_ in II–IV Q: 10.2–31.6 μg/m^3^.	Number of nonexposed were not specified. Exposure cut-off of PM_2.5_ in I Q: <10.2 μg/m^3^.	Exposure to PM_2.5_ is correlated with TLBW (aOR 1.08, 95%CI: 1.01–1.16) and SGA (aOR 1.08, 95%CI: 1.04–1.11).
Sathyanarayana et al. (2013) [63]	1997–2005 Washington State, USA Pollutants: NO_2_, PM_2.5_ and proximity to major roads	Retrospective study n = 367,046	Number of exposed were not specified. Exposure cut-off of PM_2.5_ in II–IV Q: 9.0–30.4 μg/m^3^.	Number of nonexposed were not specified. Exposure cut-off of PM_2.5_ in I Q: <9.0 μg/m^3^.	No associations were observed between PM_2.5_ exposure and SGA occurrence.
Salihu et al. (2012) [66]	2000–2007 Tampa, Florida, USA Pollutants: PM_2.5_ and PM_10_,	Retrospective study n = 12,356	8791 pregnant women exposed with PM_2.5_, PM_10_ above the median. Exposure above the median: >10.97 μg/m^3^ PM_2.5_.	3565 pregnant women exposed with PM_2.5_, PM_10_ below the median. Exposure below the median: <10.97 μg/m^3^ PM_2.5_.	Women exposed to air particulate pollutants were at elevated risk for TLBW (aOR 1.24, 95%CI: 1.07–1.43) and VLBW (aOR 1.58, 95%CI: 1.09–2.29). Exposure to PM_2.5_ was related to TLBW occurrence (aOR 1.15, 95%CI: 1.01–1.31).
Salihu et al. (2012) [67]	2000–2007 Tampa, Florida, USA Pollutants: PM_2.5_ and PM_10_	Retrospective study n = 103,961	24,090 pregnant women exposed with PM_2.5_, PM_10_ above the median. Exposure above the median: >11.28 μg/m^3^ PM_2.5_	79,871 pregnant women exposed with PM_2.5_, PM_10_ below the median. Exposure below the median: <11.28 μg/m^3^ PM_2.5_	Exposed women had increased odds for low birth weight and very low birth weight, with the greatest risk being that for very low birth weight (aOR 1.27, 95%CI 1.08–1.49). Exposure to PM_2.5_ was related to TLBW occurrence (aOR 1.07, 95%CI: 1.01–1.12). Exposure to PM_2.5_ was related to SGA occurrence (aOR 1.06, 95%CI: 1.01–1.11).
Madsen et al. (2010) [71]	1999–2002 Oslo, Norway Pollutants: NO_2_, PM_10_ and PM_2.5_	Retrospective study n = 25,229	18,921 pregnant women exposed with NO_2_, PM_10_, PM_2.5_ II–IV Q. Exposure cut-off of PM_2.5_ in II–IV Q: >9.8 μg/m^3^.	6308 pregnant women exposed with NO_2_, PM_10_, PM_2.5_ in I Q. Exposure cut-off of PM_2.5_ in I Q PM_2.5_: <9.7μg/m^3^.	No associations were observed between NO_2_, PM_10_, or PM_2.5_ exposure and SGA or TLBW occurrence.
Brauer et al. (2008) [74]	1999–2002 Vancouver, Canada Pollutants: NO, NO_2_, PM_2.5_, O_3_, proximity to major roads	Retrospective study n = 70,249	Number of exposed were not specified. Exposure cut-off of PM_2.5_ in II–IV Q: Cut-off point of exposure not specified in study.	Number of nonexposed were not specified. Exposure cut-off of PM_2.5_ in I Q PM_2.5_: Cut-off point of exposure not specified in study.	50 m distance to highways is related to increased SGA occurrence (OR 1.26, 95%CI: 1.07–1.49) and TLBW (OR 1.11, 95%CI: 1.01–1.23). Exposure to NO, NO_2_, PM_2.5_ is correlated with SGA.

**Table 4:** Characteristics of the included studies about the influence of nitrogen substances (Nitrogen oxides (NO_x_): nitric oxide—NO and nitrogen dioxide—NO_2_).

Study	Time and Place of Exposure Type of Pollutant	Character of the Study and Number of Included Patients			Outcomes
			Study Group	Control Group	
Mitku et al. (2023) [24]	2013–2017 Durban, South Africa Pollutants: PM_2.5_, SO_2_ and NO_x_ (NO and NO_2_)	Retrospective study n = 656 from low socioeconomic neighbourhoods	Number of exposed were not specified. Exposure cut-offs of NO_x_ in II–IV Q: Cut-off point of exposure not specified in study.	Number of nonexposed were not specified. Exposure cut-offs of NO_x_ in I Q: Cut-off point of exposure not specified in study.	Paradoxically decreased level of SGA after NO_x_ exposure was shown (aOR 0.9, 95%CI: 0.93–0.95).
Zhou et al. (2023) [26]	2015–2020 Chongqing, China Pollutants: PM_2.5_, PM_10_, NO_2_, CO and O_3_	Retrospective study n = 572,106	Number of exposed were not specified. Exposure cut-offs of NO_x_ in II–IV Q NO_2_: 35.5–68.2 μg/m^3^	Number of nonexposed were not specified. Exposure cut-offs of NO_2_ in I Q: 10.8–35.5 μg/m^3^	NO_2_ exposure is related to VLBW occurrence in the first (RR 1.11, 95%CI: 1.02–1.22), and second trimesters (RR 1.15, 95%CI: 1.04–1.27).
Gan et al. (2022) [28]	2017–2018 Guangzhou, China Pollutants: PM_2.5_, NO_2_, SO_2_, O_3_, and PM_10_	Prospective study n = 916	Number of exposed were not specified. Exposure cut-offs of NO_2_ in II–IV Q: Cut-off point of exposure not specified in study.	Number of nonexposed were not specified. Exposure cut-offs of NO_2_ in I Q: Cut-off point of exposure not specified in study.	TLBW is associated with maternal exposure to SO_2_ and NO_2_ (OR1.26, 95%CI: 1.05–1.51).
Huang et al. (2022) [30]	2015–2016 Wen Zhou, China Pollutants: PM_2.5_, PM_10_, SO_2_, NO_2_, and O_3_	Retrospective study n = 213,959	Number of exposed were not specified. Exposure cut-offs of NO_x_ in II–IV Q: 40.1–52.9 μg/m^3^	Number of nonexposed were not specified. Exposure cut-offs of NO_x_ in I Q: <40.1 μg/m^3^	TLBW is associated with maternal exposure to NO_2_ (aOR 1.13, 95%CI: 1.01–1.26) during the entire pregnancy. A significant influence was shown, especially in the 2nd trimester.
Shang et al. (2021) [36]	2015–2018 Xi’an city of Shaanxi, China Pollutants: high level of air quality index (AQI), PM_2.5_, PM_10_, SO_2_, CO, O_3_, NO_2_	Retrospective study n = 321,521	Number of exposed were not specified. Exposure cut-offs of NO_2_ in II–IV Q: >45.9 μg/m^3^	Number of nonexposed were not specified. Exposure cut-offs of NO_2_ in I Q: <45.9 μg/m^3^	No associations were observed between NO_2_ exposure and TLBW occurrence.
Bergstra et al. (2021) [38]	2012–2017 Netherlands Pollutants: PM_10_, NO_x_, SO_2_, and volatile organic compounds (VOC)	Cross-sectional study n = 4488	Number of exposed were not specified. Exposure cut-offs of NO_x_ in II–IV Q: 1.65–9.50 μg/m^3^	Number of nonexposed were not specified. Exposure cut-offs of NO_x_ in I Q: 0.49–1.65 μg/m^3^	TLBW is associated with maternal exposure to NO_x_ (OR 1.20, 95%CI: 1.06–1.35).
Nobles et al. (2019) [43]	2002–2010 20 hospitals in USA Pollutants: SO_2_, O_3_, NO_x_, NO_2_, CO, PM_10_ and PM_2.5_	Retrospective study n = 109,126 births	Number of exposed were not specified. Exposure cut-offs of NO_2_, and NO_x_ in II–IV Q:Cut-off point of exposure not specified in study.	Number of nonexposed were not specified. Exposure cut-offs of NO_2_, and NO_x_ in I Q: Cut-off point of exposure not specified in study.	Risk of SGA increases in the third trimester every 10th percentile per interquartile increase in exposure of: NO_x_ (RR 1.08, 95%CI:1.03–1.14) NO_2_ (RR 1.05, 95%CI: 1.01–1.10).
Dedele et al. (2017) [48]	2008 Kaunas, Lithuania Pollutant: NO_2_	Retrospective study n = 3292	2146 pregnant women exposed with NO_2_. Exposure cut-offs of NO_2_ in II–III Tertiles (T) II T 20–24 μg/m^3^ III T >24 μg/m^3^	1146 pregnant women nonexposed with NO_2_. Exposure cut-off of NO_2_ in I T <19 μg/m^3^	Increased maternal exposure (III T) to NO_2_ tended to increase the risk for TLBW (aOR 1.89, 95%CI: 1.05–3.43).
Capobussi et al. (2016) [49]	2005–2012 Como, Italy Pollutants: NO_x_, NO_2_, SO_2_, O_3_, CO and PM_10_	Retrospective study n = 27,128	Number of exposed were not specified. Exposure cut-offs of NO_2_, and NO_x_ in II–IV Q: Cut-off point of exposure not specified in study.	Number of nonexposed were not specified. Exposure cut-offs of NO_2_, and NO_x_ in I Q: Cut-off point of exposure not specified in study.	Women exposed to NO_x_ in the third trimester had a higher risk having a SGA baby (aOR 1.12, 95%CI 1.01–1.27)
Stieb et al. (2016) [50]	1999–2008 Canada Pollutants: PM_2.5_ and NO_2_	Retrospective study n = 2,928,515	Number of exposed were not specified. Exposure cut-offs of NO_2_ II–IV Q: 7.00 ≥ 18.52 μg/m^3^.	Number of nonexposed were not specified. Exposure cut-offs of NO_2_ in I Q: <7.00 μg/m^3^.	SGA occurrence is related to every 20 ppb NO_2_ exposure (OR 1.04, 95%CI: 1.02–1.06) and TLBW related to every 20 ppb NO_2_ exposure in 16.2 g reduction, 95%CI: 13.6–18.8 g.
Lavigne et al. (2016) [52]	2005–2012 Ontario, Canada Pollutants: PM_2.5_, NO_2_, and O_3_	Retrospective study n = 818,400	Number of exposed were not specified. Exposure cut-offs of >5 percentile of NO_2_ (>6 ppb)	Number of nonexposed were not specified. Exposure cut-offs of ≤5 percentile of NO_2_ (≤6 ppb)	No associations were observed between PM_2.5_, NO_2_, or O_3_ exposure and SGA or TLBW occurrence.
Hannam et al. (2014) [57]	2004–2008 Northwest England, UK Pollutants: NO_x_, NO_2_, CO, PM_2.5_ and PM_10_	Retrospective study n = 203,562	Number of exposed were not specified. Exposure cut-offs of NO_2_, and NO_x_ in II–IV Q: II–IV Q NO_x_ (96.0 ≥ 225.9 μg/m^3^) II–IV Q NO_2_ (63.6 ≥ 169.7 μg/m^3^)	Number of nonexposed were not specified. Exposure cut-offs of NO_2_, and NO_x_ in I Q: I Q NO_x_ (13.0–55.4 μg/m^3^) I Q NO_2_ (8.6–42.9 μg/m^3^)	NO_x_, NO_2_, CO, PM_2.5_, PM_10_ is related with increased risk of SGA infant. Small statistically significant association was observed for PM_10_ and SGA, particularly with exposure in the first and third trimesters. Similar effects on SGA were also found for NO_2_ (aOR 1.66, 95%CI: 1.47–1.87) in later pregnancy, but no overall increased risk was observed.
Olsson et al. (2013) [62]	1997–2006 Stockholm, Sweden Pollutants: O_3_ and NO_x_	Retrospective study n = 120,755	Number of nonexposed were not specified. Exposure cut-offs of NO_x_ in II–IV Q: Cut-off point of exposure not specified in study.	Number of nonexposed were not specified. Exposure cut-offs of NO_x_ in I Q: Cut-off point of exposure not specified in study.	No associations were observed between O_3_ or NO_x_ exposure and SGA or TLBW occurrence.
Sathyanarayana et al. (2013) [63]	1997–2005 Washington State, USA Pollutants: NO_2_, PM_2.5_ and proximity to major roads	Retrospective study n = 367,046	Number of exposed were not specified. Exposure cut-offs of NO_2_ in II–IV Q: 12.4–36.8 μg/m^3^	Number of nonexposed were not specified. Exposure cut-offs of NO_2_ in I Q: <12.4 μg/m^3^	SGA births with increasing quartile of first trimester NO_2_ exposure in: II Q (OR 1.01, 95%CI: 0.97–1.04), III Q (OR 1.06, 95%CI: 1.03–1.10), and IV Q (OR 1.08, 95%CI: 1.04–1.12). No associations were observed between PM_2.5_ exposure and SGA occurrence.
Le et al. (2012) [64]	1990–2001 Detroit, Michigan, USA Pollutants: CO, NO_2_, PM_10_ and O_3_	Retrospective study n = 164,905	Number of exposed were not specified. Exposure cut-offs of NO_x_ in II–IV Q: >6.8 ppb	Number of nonexposed were not specified. Exposure cut-offs of NO_x_ in I Q: <6.8 ppb	SGA was associated with exposure of NO_2_ (aOR 1.11, 95%CI: 1.03–1.21) in first month.
van den Hooven et al. (2012) [65]	2001–2005 Rotterdam, Netherlands Pollutants: PM_10_ and NO_2_	Prospective study n = 7772	6928 pregnant women exposed with NO_2_. Quartiles of exposure cut-offs in II–IV Q: II–IV Q NO_2_ (37.2–56.9 μg/m^3^)	844 pregnant women exposed with NO_2_. Quartiles of exposure cut-offs in I Q: I Q NO_2_ (<37.2 μg/m^3^)	No associations were observed between NO_2_ exposure and SGA or TLBW occurrence.
Malmqvist et al. (2011) [68]	1999–2005 Scania (Skåne), Sweden Pollutant: NO_x_	Retrospective study n = 81,110	60,530 pregnant women exposed with NO_x_ in II–IV Q. Exposure cut-offs of NO_x_ in: II Q (9.0–14.1 μg/m^3^) III Q (14.2–22.6 μg/m^3^) IV Q (>22.7 μg/m^3^)	20,580 pregnant women exposed with NO_x_ in I Q. Exposure cut-offs of NO_x_ in I Q: 2.5–8.9 μg/m^3^	NO_x_ exposure is related with SGA (I vs. IV) (OR 1.12, 95%CI: 1.01–1.24)
Kashima et al. (2011) [69]	1997–2008 Shizuoka, Japan Pollutants: distance to a major road, distance-weighted traffic density (DWTD) and NO_2_	Retrospective study n = 14,204	Number of exposed were not specified. Exposure to a distance to a major road (<200 m), DWTD and mean NO_2_ concentration across roadside stations 51.8 ± 7.5 μg/m^3^ Exposure cut-offs in NO_2_ in II–IV Q: First 3 months (12.2–34.7 μg/m^3^) Last 3 months (12.0–35.7 μg/m^3^)	Number of nonexposed were not specified. Exposure to a distance to a major road (≥200 m), DWTD and mean NO_2_ concentration across general stations 30.09 ± 6.2 μg/m^3^ Exposure cut-off in I Q NO_2_: First 3 months (8.7–12.2 μg/m^3^) Last 3 months (6.3–12.0 μg/m^3^)	0.6 g (95%CI: −1.8–0.6 g) birth weight reduction is following to every 500 m decrease of the distance to a major road with breakpoint at 2200 m distance and the higher SGA occurrence by distance ≤ 624 m. No associations were observed between NO_2_ exposure and SGA or TLBW occurrence.
Gehring et al. (2011) [70]	2003–2004 Amsterdam, Netherlands Pollutants: NO_2_, proximity to major roads (<50 m)	Prospective study n = 7762	Number of exposed were not specified. Exposure cut-offs of NO_x_ in: II Q NO_2_ (34.6–37.4 μg/m^3^) III Q NO_2_ (37.4–40.2 μg/m^3^) IV Q NO_2_ (>40.2 μg/m^3^)	Number of nonexposed were not specified. Exposure cut-offs of NO_x_ in I Q: <34.6 μg/m^3^	No associations were observed between NO_2_ exposure and SGA or TLBW occurrence.
Madsen et al. (2010) [71]	1999–2002 Oslo, NorwayPollutants: NO_2_, PM_10_, and PM_2.5_	Retrospective study n = 25,229	18,921 pregnant women exposed with NO_2_, PM_10_, PM_2.5_ II–IV Q. Exposure cut-offs of NO_2_ in II–IV Q: >20.4 μg/m^3^	6308 pregnant women exposed with NO_2_, PM_10_, PM_2.5_ in I Q. Exposure cut-offs of NO_2_ in I O: <20.3 μg/m^3^	No associations were observed between NO_2_, PM_10_, or PM_2.5_ exposure and SGA or TLBW occurrence.
Ballester et al. (2010) [72]	2003–2005 Valencia, Spain Pollutants: NO_2_	Retrospective study n = 785	Number of exposed were not specified. Exposure cut-offs of NO_2_ in II–IV Q: >27.3 μg/m^3^	Number of nonexposed were not specified. Exposure cut-offs of NO_2_ in I O: <27.3 μg/m^3^	10 μg/m^3^ increase in NO_2_ exposure in the second trimester is related with SGA (OR 1.37, 95%CI: 1.01–1.85). >40 μg/m3 NO_2_ exposure in the first trimester was associated with a change in birth weight of −40.3 g, 95%CI: −96.3–15.6 g).
Brauer et al. (2008) [74]	1999–2002 Vancouver, Canada Pollutants: NO, NO_2_, PM_2.5_, O_3_ and proximity to major roads	Retrospective study n = 70,249	Number of exposed were not specified. Exposure cut-offs of NO, and NO_2_, in II–IV Q:Cut-off point of exposure not specified in study.	Number of nonexposed were not specified. Exposure cut-offs of NO, and NO_2_, in I Q: Cut-off point of exposure not specified in study.	50 m distance to highways is related to increased SGA occurrence (OR 1.26, 95%CI: 1.07–1.49) and TLBW (OR 1.11, 95%CI: 1.01–1.23). Exposure to NO, NO_2_, PM_2.5_ is correlated with SGA. 10 μg/m^3^ increase of NO exposure is related with 5 % increased SGA occurrence (OR 1.05, 95%CI: 1.03–1.08).
Hansen et al. (2007) [75]	2000–2003 Brisbane, Australia Pollutants: PM_10_, NO_2_ and O_3_	Retrospective study n = 26,617	Number of exposed were not specified. Exposure cut-offs of NO_2_ in II–IV Q: 5.5–24.2 ppb	Number of nonexposed were not specified. Exposure cut-offs of NO_2_ in I Q: <5.5 ppb	No associations were observed between PM_10_, NO_2_, or O_3_ exposure and SGA or TLBW occurrence.
Lin et al. (2004) [79]	1995–1997 Taipei and Kaohsiung, Taiwan Pollutants: SO_2_, PM_10_, CO, O_3_ and NO_2_	Retrospective study n = 31,530 (Kaohsiung) n = 60,758 (Taipei)	31,530 pregnant women from Kaohsiung exposed with mean concentration of NO_2_ was similar in both groups.	60,758 pregnant women from Taipei exposed with mean concentration of NO_2_ was similar in both groups.	Exposure with NO_2_ was similar in both groups.
Lin et al. (2004) [80]	1995–1997 Taipei and Kaohsiung, Taiwan Pollutants: SO_2_, PM_10_, CO, O_3_ and NO_2_	Retrospective study n = 92,288	Number of exposed were not specified. Exposure cut-offs of NO_2_ in II–IV Q: >26.1 ppm	Number of nonexposed were not specified. Exposure cut-offs of NO_2_ in I Q: <26.1 ppm	No associations were observed between PM_10_, CO, O_3_, or NO_2_ exposure and TLBW occurrence.
Lee et al. (2003) [81]	1996–1998 Seoul, Korea Pollutants: CO, PM_10_, SO_2_ and NO_2_	Retrospective study n = 388,105	Number of exposed were not specified. Exposure cut-offs of NO_2_ in II–IV Q: 25.0–65.1 ppb	Number of nonexposed were not specified. Exposure cut-offs of NO_2_ in I Q: 10.2–25.0 ppb	Second-trimester exposure to NO_2_ increased the risk for TLBW (aOR 1.03, 95%CI: 1.01–1.06). CO, PM_10_, SO_2_ and NO_2_ during 1–2 trimesters is related with TLBW.
Lin et al. (2001) [86]	1993–1996 Lin-Yuan and Taicei, Taiwan Pollutants: SO_2_, NO_2_, PM_10_, SO_4_^2−^, NH_4_^+^ and NO_3_^−^	Retrospective study n = 2545	1677 pregnant women from Lin-Yuan municipality. Exposure cut-offs in II–IV Q: NO_2_ (12.1 ± 2.2 ppb), NO_3_^−^ (124.7 ± 1.9 nmol/m^3^)	868 pregnant women from Taicei municipality. Exposure cut-offs in I Q: NO_2_ (8.6 ± 1.4 ppb), NO_3_^−^ (103.9 ± 2.0 nmol/m^3^)	Higher exposure of SO_2_, NO_2_, PM_10_, SO_4_^2−^, NO_3_^−^, petrochemical municipality in Lin-Yuan leads to 3.22% TLBW occurrence in comparison to lower exposure in control municipality Taicei which lead to 1.84% TLBW occurrence.

**Table 5:** Characteristics of the included studies about the influence of ozone (O_3_).

Study	Time and Place of Exposure Type of Pollutant	Character of the Study and Number of Included Patients			Outcomes
			Study Group	Control Group	
Zhou et al. (2023) [26]	2015–2020 Chongqing, China Pollutants: PM_2.5_, PM_10_, NO_2_, CO and O_3_	Retrospective study n = 572,106	Number of exposed were not specified. Ex Exposure cut-offs of O_3_ in II–IV Q: 30.2–105.7 μg/m^3^	Number of nonexposed were not specified. Exposure cut-offs of O_3_ in I Q: 8.3–30.2 μg/m^3^	O_3_ exposure is related with VLBW occurrence in the entire pregnancy (RR 1.08, 95%CI: 1.01–1.15), and in the second trimester (RR 1.08, 95%CI: 1:02–1.14).
Gan et al. (2022) [28]	2017–2018 Guangzhou, China Pollutants: PM_2.5_, NO_2_, SO_2_, O_3_, and PM_10_	Prospective study n = 916	Number of exposed were not specified. Exposure cut-offs of O_3_ in II–IV Q: Cut-off point of exposure not specified in study.	Number of nonexposed were not specified. Exposure cut-offs of O_3_ in I Q: Cut-off point of exposure not specified in study.	TLBW is associated with maternal exposure to SO_2_ and O_3_ (OR 1.24, 95%CI: 1.05–1.48).
Huang et al. (2022) [30]	2015–2016 Wen Zhou, China Pollutants: PM_2.5_, PM_10_, SO_2_, NO_2_, and O_3_	Retrospective study n = 213,959	Number of exposed were not specified. Exposure cut-offs of O_3_ in II–IV Q: 83.6–102.4 μg/m^3^	Number of nonexposed were not specified. Exposure cut-offs of O_3_ in I Q: <83.6 μg/m^3^	No associations were observed between O_3_ exposure and TLBW occurrence. Moreover, O_3_ seems to have positive impact on Macrosomia occurrence.
Shang et al. (2021) [36]	2015–2018 Xi’an city of Shaanxi, China Pollutants: high level of air quality index (AQI), PM_2.5_, PM_10_, SO_2_, CO, O_3_ and NO_2_	Retrospective study n = 321,521	Number of exposed were not specified. Exposure cut-offs of O_3_ in II–IV Q: >43.6 μg/m^3^	Number of nonexposed were not specified. Exposure cut-offs of O_3_ in I Q: <43.6 μg/m^3^	Exposure of O_3_ is associated with increased term birth weight (β 4.15, 95%CI: 3.49–4.81) and macrosomia (OR 1.02, 95%CI: 1.017–1.03).
Wang et al. (2021) [37]	2015–2017 Guangzhou, China Pollutant: O_3_	Retrospective study n = 444,096	Number of exposed were not specified. Exposure with 1-h maximum O_3_ lever within a day 84.5–112.9 μg/m^3^	Number of nonexposed were not specified. Exposure with 8-h maximum O_3_ lever within a 73–90 μg/m^3^	Maximal 1 h exposure to higher level of during O_3_ the whole pregnancy (aOR 1.3, 95%CI: 1.06–1.58), especially in second trimester (aOR 1.21, 95%CI: 1.07–1.36) and maximal 8 h exposure to slightly lower level of O_3_ (aOR 1.24, 95%CI: 1.01–1.52), and in second trimester (aOR 1.17, 95%CI: 1.03–1.33) are associated with higher risk of TLBW.
Nobles et al. (2019) [43]	2002–2010 20 hospitals in USA Pollutants: SO_2_, O_3_, NO_x_, NO_2_, CO, PM_10_ and PM_2.5_	Retrospective study n = 109,126 births	Number of exposed were not specified. Exposure cut-offs of O_3_ in II–IV Q: Cut-off point of exposure not specified in study.	Number of nonexposed were not specified. Exposure cut-offs of O_3_ in I Q: Cut-off point of exposure not specified in study.	O_3_ exposure in the third trimester is associated with a lower risk of SGA (RR 0.95, 95%CI: 0.92–0.97).
Costa Nascimento et al. (2017) [47]	2012–2013 São José do Rio Preto, Brazil Pollutants: NO_2_, PM_10_ and O_3_	Retrospective longitudinal study n = 8948	Number of exposed were not specified. Exposure cut-offs of O_3_ in II–IV Q: 52.36–81.98 μg/m^3^	Number of nonexposed were not specified. Exposure cut-offs of O_3_ in I Q: <52.36 μg/m^3^	Exposure to O_3_ was significantly associated with TLBW after 90 days of exposure (aOR = 1.48, 95%CI: 1.10–2.0) and after 30 days of exposure (aOR 1.38, 95%CI: 1.03–1.84).
Lavigne et al. (2016) [52]	2005–2012 Ontario, Canada Pollutants: PM_2.5_, NO_2_, and O_3_	Retrospective study n = 818,400	Number of exposed were not specified. Exposure cut-offs of >5 percentile of O_3_ (>23 ppb)	Number of nonexposed were not specified. Exposure cut-offs of ≤5 percentile of O_3_ (≤23 ppb)	No associations were observed between PM_2.5_, NO_2_, or O_3_ exposure and SGA or TLBW occurrence.
Brown et al. (2015) [53]	2001–2006 New York, USA Pollutants: O_3_ and PM_2.5_	Retrospective study n = 480,430	Number of exposed were not specified. Exposure cut-offs of O_3_ in II–IV Q: 35.62–60.35 ppb	Number of exposed were not specified. Exposure cut-offs of O_3_ in I Q: 15.52–35.61 ppb	There was paradoxical effect of decreased SGA occurrence after exposure of III Q of O_3_ (aOR 0.86, 95%CI: 0.81–0.92).
Vinikoor-Imler et al. (2014) [58]	2003–2005 North Carolina, USA Pollutants: PM_2.5_ and O_3_	Retrospective study n = 312,638	Number of exposed were not specified. Exposure cut-offs of O_3_ in II–IV Q: Cut-off point of exposure not specified in study.	Number of nonexposed were not specified. Exposure cut-offs of O_3_ in I Q: Cut-off point of exposure not specified in study.	Exposure to O_3_ is correlated with SGA (aOR 1.16, 95%CI: 1.11–1.22) and TLBW (aOR 2.03, 95%CI: 1.80–2.30).
Olsson et al. (2013) [62]	1997–2006 Stockholm, Sweden Pollutants: O_3_ and NO_x_	Retrospective study n = 120,755	Number of exposed were not specified. Exposure cut-offs of O_3_ in II–IV Q: Cut-off point of exposure not specified in study.	Number of nonexposed were not specified. Exposure cut-offs of O_3_ in I Q: Cut-off point of exposure not specified in study.	No associations were observed between O_3_ or NO_x_ exposure and SGA or TLBW occurrence.
Le et al. (2012) [64]	1990–2001 Detroit, Michigan, USA Pollutants: CO, NO_2_, PM_10_ and O_3_	Retrospective study n = 164,905	Number of exposed were not specified. Exposure cut-offs of O_3_ in II–IV Q: >92 ppb O_3_	Number of nonexposed were not specified. Exposure cut-offs of O_3_ in I Q: <92 ppb	SGA was associated with exposure to O_3_ in third trimester (aOR 1.11, 95%CI: 1.02–1.20).
Nascimento and Moreira (2009) [73]	2001 São José dos Campos, Brazil Pollutants: SO_2_, O_3_ and PM_10_	Retrospective study n = 2529	Number of exposed were not specified. Exposure cut-offs of O_3_ in II–IV Q: Cut-off point of exposure not specified in study.	Number of nonexposed were not specified. Exposure cut-offs of O_3_ in I Q: Cut-off point of exposure not specified in study.	O_3_ showed borderline statistical significance in third quartile, with an increase of nearly 100% in the odds of TLBW (aOR 1.26, 95%CI: 1.00–1.58).
Brauer et al. (2008) [74]	1999–2002 Vancouver, Canada Pollutants: NO, NO_2_, PM_2.5_, O_3_ and proximity to major roads	Retrospective study n = 70,249	Number of exposed were not specified. Exposure cut-offs of O_3_ in II–IV Q: Cut-off point of exposure not specified in study.	Number of nonexposed were not specified. Exposure cut-offs of O_3_ in I Q: Cut-off point of exposure not specified in study.	50 m distance to highways is related to increased SGA occurrence (OR 1.26, 95%CI: 1.07–1.49) and TLBW (OR 1.11, 95%CI: 1.01–1.23). No associations were observed between O_3_ exposure and SGA occurrence was shown.
Hansen et al. (2007) [75]	2000–2003 Brisbane, Australia Pollutants: PM_10_, NO_2_ and O_3_	Retrospective study n = 26,617	Number of exposed were not specified. Exposure cut-offs of O_3_ in II–IV Q: 21.0–61.1 ppb	Number of nonexposed were not specified. Exposure cut-offs of O_3_ in I Q: <21.0 ppb	No associations were observed between O_3_ exposure and SGA or TLBW occurrence.
Lin et al. (2004) [79]	1995–1997 Taipei and Kaohsiung, Taiwan Pollutants: SO_2_, PM_10_, CO, O_3_ and NO_2_	Retrospective study n = 31,530 (Kaohsiung) n = 60,758 (Taipei)	31,530 pregnant women from Kaohsiung exposed with mean concentration of O_3_ (29.4–49.5 ppm)	60,758 pregnant women from Taipei exposed with mean concentration of O_3_ (14.1–20.4 ppm)	Higher exposure of SO_2_, PM_10_, CO, O_3_, NO_2_ in Kaohsiung leads to 13% higher TLBW occurrence than lower exposure in Taipei (OR 1.13, 95%CI: 1.03–1.24).
Lin et al. (2004) [80]	1995–1997 Taipei and Kaohsiung, Taiwan Pollutants: SO_2_, PM_10_, CO, O_3_ and NO_2_	Retrospective study n = 92,288	Number of exposed were not specified. Exposure cut-offs of O_3_ in II–IV Q: >19.6 ppm	Number of nonexposed were not specified. Exposure cut-offs of O_3_ in I Q: <19.6 ppm	No associations were observed between PM_10_, CO, O_3_, or NO_2_ exposure and TLBW occurrence.
Chen et al. (2002) [84]	1991–1999 Nevada State, USA Pollutants: PM_10_, CO and O_3_	Retrospective study n = 39,338	32,682 pregnant women exposed with O_3_ at the third trimester (>17.93 ppb)	3623 pregnant women with low exposure to O_3_ at the third trimester (<17.93 ppb)	O_3_ exposure was found not to be related to birth weight.

**Table 6:** Characteristics of the included studies about the influence of sulfur dioxide (SO_2_), carbon monoxide (CO), and volatile organic compounds (VOCs).

Study	Time and Place of Exposure Type of Pollutant	Character of the Study and Number of Included Patients			Outcomes
			Study Group	Control Group	
Mitku et al. (2023) [24]	2013–2017 Durban, South Africa Pollutants: PM_2.5_, SO_2_ and NO_x_ (NO and NO_2_)	Retrospective study n = 656 from low socioeconomic neighbourhoods	Number of exposed were not specified. Exposure with SO_2_ in II–IV Q: Cut-off point of exposure not specified in study.	Number of nonexposed were not specified. Exposure with PM_2.5_, SO_2_ and NO_x_ in I Q: Cut-off point of exposure not specified in study.	Increased SGA occurrence risk is associated with exposure to SO_2_ (aOR 1.1, 95%CI: 1.01–1.13).
Zhang et al. (2023) [25]	2017–2021 Wuhan, China Pollutants: Air Pollution Score (APS)–6 pollutants assessed simultaneously (PM_2.5_, PM_10_, NO_2_, CO, O_3_ and SO_2_)	Retrospective study n = 31,283	Number of exposed were not specified. Exposure with APS in II-V Quintile. Cut-off point of exposure not specified in study.	Number of nonexposed were not specified. Exposure with APS in I Quintile. Cut-off point of exposure not specified in study.	APS exposure in second trimester is related to SGA (OR 1.43, 95%CI: 1.23–1.65) and during the entire pregnancy (OR 1.35, 95%CI: 1.16–1.56). APS exposure increased 2.5% risk of SGA for each 10 μg/m^3^ elevated (aOR 1.025, 95%CI: 1.005–1.046).
Zhou et al. (2023) [26]	2015–2020 Chongqing, China Pollutants: PM_2.5_, PM_10_, NO_2_, CO, SO_2_ and O_3_	Retrospective study n = 572,106	Number of exposed were not specified. Exposure with CO and SO_2_ in II–IV Q: II–IV Q CO (0.89–1.52 mg/m^3^) II–IV Q SO_2_ (7.3–22.1 μg/m^3^)	Number of nonexposed were not specified. Exposure with CO and SO_2_ in I Q: I Q CO (0.54–0.89 mg/m^3^) I Q SO_2_ (3.2–7.3 μg/m^3^)	No association between CO and SO_2_ and SGA or LBTW was shown.
Gan et al. (2022) [28]	2017–2018 Guangzhou, China Pollutants: PM_2.5_, NO_2_, SO_2_, O_3_, and PM_10_	Prospective study n = 916	Number of exposed were not specified. Exposure with SO_2_ in II–IV Q: Cut-off point of exposure not specified in study.	Number of nonexposed were not specified. Exposure with SO_2_ in I Q: Cut-off point of exposure not specified in study.	TLBW is associated with maternal exposure to: SO_2_ and NO_2_ (OR1.26, 95%CI: 1.05–1.51) SO_2_ and O_3_ (OR 1.24, 95%CI: 1.05–1.48) SO_2_ and PM_2.5_ (OR 1.28, 95%CI: 1.07–1.52) SO_2_ and PM_10_ (OR 1.23, 95%CI: 1.03–1.46)
Gong and Zhan (2022) [29]	1996–2008 Texas, USA Pollutants: benzaldehyde, sum of Photochemical Assessment Monitoring Stations (PAMS) target compounds, n-undecane, m-tolualdehyde, organic carbon fraction 2 (OC2), ethylene dibromide, valeraldehyde, propionaldehyde, 4-methyl-1-pentene, and zirconium	Retrospective study n = 470,684	Exposure cut-offs of: Benzaldehyde > 0.04 ppbv (n = 187) Sum of PAMS target compounds > 151.11 ppbC (n = 155) n-Undecane > 0.01 ppbv (n = 250) m-Tolualdehyde > 0.01 ppbv (n = 181) OC2 >0.83 μg/m^3^ (n = 235) Ethylene dibromide > 0.00 ppbv (n = 84) Valeraldehyde > 0.03 ppbv (n = 206) Propionaldehyde > 0.17 ppbv (n = 173) 4-Methyl-1-Pentene > 0.00 ppbv (n = 400) Zirconium PM_2.5_ LC > 0.00 µg/m^3^ (n= 220)	Exposure cut-offs of: Benzaldehyde 0.04 < ppbv (n = 162) Sum of PAMS target compounds < 151.11 ppbC (n = 134) n-Undecane < 0.01 ppbv (n = 240) m-Tolualdehyde < 0.01 ppbv (n = 161) OC2 0.83 μg/m^3^ (n = 208) Ethylene dibromide = 0.00 ppbv (n = 1684) Valeraldehyde < 0.03 ppbv (n = 191) Propionaldehyde < 0.17 ppbv (n = 159) 4-Methyl-1-Pentene = 0.00 ppbv (n = 2027) Zirconium PM_2.5_ LC = 0.00 µg/m^3^ (n = 203)	TLBW is associated with maternal exposure to: Benzaldehyde (aOR 2.66, 95%CI: 1.38–5.12) Sum of PAMS target compounds (aOR 2.02, 95%CI: 1.08–3.78) n-Undecane (aOR 2.04, 95%CI: 1.22–3.40) m-Tolualdehyde (aOR 2.02, 95%CI: 1.05–3.89) OC2 (aOR 1.98, 95%CI: 1.21–3.26) Valeraldehyde (aOR 1.96, 95%CI: 1.14–3.38) Propionaldehyde (aOR 1.92, 95%CI: 1.01–3.65) Ethylene dibromide (aOR 1.97, 95%CI: 1.24–3.15) 4-Methyl-1-Pentene (aOR 1.44, 95%CI: 1.14–1.82) Zirconium PM_2.5_ LC (aOR 1.88, 95%CI: 1.02–3.45)
Huang et al. (2022) [30]	2015–2016 Wen Zhou, China Pollutants: PM_2.5_, PM_10_, SO_2_, NO_2_, and O_3_	Retrospective study n = 213,959	Number of exposed were not specified. Exposure with SO_2_ in II–IV Q: 13.3–19.3 μg/m^3^	Number of nonexposed were not specified. Exposure with SO_2_ in I Q: <13.3 μg/m^3^	TLBW is associated with maternal exposure to SO_2_ during the entire pregnancy (aOR 1.32, 95%CI: 1.07–1.64). The significant influence was shown especially in the 2nd trimester.
Shen et al. (2022) [32]	2015–2016 24 provinces in China Pollutants: PM_2.5_, CO, NH_4_^+^ (ammonium), and SO_4_^2−^ (sulphate)	Retrospective study n = 70,206	Number of exposed were not specified. Exposure cut-offs of II–IV Q: CO (8–31 μg/m^3^) NH_4_^+^ (7–16 μg/m^3^) SO_4_^2−^ (12–24 μg/m^3^)	Number of nonexposed were not specified. Exposure cut-offs of I Q: CO (<8 μg/m^3^) NH_4_^+^ (<7 μg/m^3^) SO_4_^2−^ (<12 μg/m^3^)	PM_2.5_ exposure during pregnancy is associated with 16%, 95%CI: 3–30% higher risk of SGA. SGA is also associated with maternal exposure to: CO (OR 1.15, 95%CI: 1.00–1.32), NH_4_^+^ (OR 1.12, 95%CI: 1.01–1.25), and SO_4_^2−^ (OR 1.12, 95%CI: 1.04–1.21)
Shang et al. (2021) [36]	2015–2018 Xi’an city of Shaanxi, China, Pollutants: high level of air quality index (AQI), PM_2.5_, PM_10_, SO_2_, CO, O_3_ and NO_2_	Retrospective study n = 321,521	Number of exposed were not specified. Exposure cut-offs of II–IV Q: II–IV Q AQI (>66.2) II–IV Q SO_2_ (>11.1 μg/m^3^) II–IV Q CO (>1.3 μg/m^3^)	Number of nonexposed were not specified. Exposure cut-offs of I Q: I Q AQI (<66.2) I Q SO_2_ (<11.1 μg/m^3^) I Q CO (<1.3 μg/m^3^)	TLBW is associated with maternal exposure to: AQI (OR 1.02, 95%CI: 1.006–1.03) SO_2_ (OR 1.03, 95%CI: 1.01–1.06) CO (OR 1.007, 95%CI: 1.001–1.014).
Bergstra et al. (2021) [38]	2012–2017 Netherlands Pollutants: PM_10_, NO_x_, SO_2_, and volatile organic compounds (VOC)	Cross-sectional study n = 4488	Number of exposed were not specified. Exposure cut-offs of II–IV Q: SO_2_ (0.63–2.33 μg/m^3^) VOC (1.31–9.04 μg/m^3^)	Number of nonexposed were not specified. Exposure cut-offs of I Q: SO_2_ (0.21–0.63 μg/m^3^) VOC (0.34–1.31 μg/m^3^)	TLBW is associated with maternal exposure to SO_2_ (OR 1.20, 95%CI: 1.0–1.43) and VOC (OR 1.21, 95%CI: 1.08–1.35).
Nobles et al. (2019) [43]	2002–2010 20 hospitals in USA Pollutants: SO_2_, O_3_, NO_x_, NO_2_, CO, PM_10_ and PM_2.5_	Retrospective study n = 109,126 births	Number of exposed were not specified. Exposure with CO and SO_2_ in II–IV Q: Cut-off point of exposure not specified in study.	Number of nonexposed were not specified. Exposure with CO and SO_2_ in I Q: Cut-off point of exposure not specified in study.	Risk of SGA increases in the third trimester every 10th percentile per interquartile increase in exposure of CO (RR 1.05, 95%CI 1.00, 1.10).
Gong et al. (2018) [45]	1996–2008 Texas, USA Pollutants: Multiple VOC (benzene, benzo(g,h,i)perylene, cumene, cyclohexane, dichloromethane, ethylbenzene, ethylene, naphthalene, n-hexane, propylene, styrene, toluene), zinc and mercury	Retrospective study n = 470,530	Number of exposed were not specified. Pregnant women in “low”, “medium” and “high” exposure group to pollution—defined by authors.	Number of nonexposed were not specified. Pregnant women in “zero” exposure group to pollution—defined by authors.	TLBW is associated with maternal exposure to: benzene (aOR 1.06, 95%CI: 1.04–1.08), benzo(g,h,i)perylene (aOR 1.04, 95%CI: 1.02–1.07), cumene (aOR 1.05, 95%CI: 1.03–1.07), cyclohexane (aOR 1.04, 95%CI: 1.02–1.07), dichloromethane (aOR 1.04, 95%CI: 1.03–1.07), ethylbenzene (aOR 1.05, 95%CI: 1.03–1.06), ethylene (aOR 1.06, 95%CI: 1.03–1.09), naphthalene (aOR 1.03, 95%CI: 1.01–1.05), n-hexane (aOR 1.06, 95%CI: 1.04–1.08), propylene (aOR 1.06, 95%CI: 1.03–1.10), styrene (aOR 1.06, 95%CI: 1.04–1.08), toluene (aOR 1.05, 95%CI: 1.03–1.07), mercury (aOR 1.04, 95%CI: 1.02–1.07), zinc (fume or dust) (aOR 1.10, 95%CI: 1.06–1.13)
Wu (2018) [46]	2013–2016 Jinan, China Pollutants: PM_2.5_, NO_2_ and SO_2_	Retrospective study n = 43,855	Number of exposed were not specified. Exposure cut-offs of SO_2_ in II–IV Q: 42.6–148.0 μg/m^3^	Number of nonexposed were not specified. Exposure cut-offs of SO_2_ in I Q: <42.5 μg/m^3^	No association of SO_2_ exposure and SGA or TLBW was shown.
Capobussi et al. (2016) [49]	2005–2012 Como, Italy Pollutants: NO_x_, NO_2_, SO_2_, O_3_, CO and PM_10_	Retrospective study n = 27,128	Number of exposed were not specified. Exposure cut-offs of CO and SO_2_ in II–IV Q: Cut-off point of exposure not specified in study.	Number of nonexposed were not specified. Exposure cut-offs of CO and SO_2_ in I Q: Cut-off point of exposure not specified in study.	No association of CO or SO_2_ exposure and SGA or TLBW was shown.
Poirier et al. (2015) [54]	2008–2012 Nova Scotia, Canada Pollutants: NO_2_, SO_2_, PM_2.5_ and PM_10_	Retrospective study n = 13,400 births in NO_2_, PM_2.5_ PM_10_, benzene, toluene group n = 12,834 births in SO_2_ group	Number of exposed were not specified. Exposure cut-offs of SO_2_ in II–IV Q: Cut-off point of exposure not specified in study.	Number of nonexposed were not specified. Exposure cut-offs of SO_2_ in I Q: Cut-off point of exposure not specified in study.	Compared with women in the I quartile of exposure to SO_2_, those in the IV quartile of exposure were positively associated with TLBW (aOR 1.52, 95%CI: 1.03, 2.26).
Hannam et al. (2014) [57]	2004–2008 Northwest England, UK Pollutants: NO_x_, NO_2_, CO, PM_2.5_ and PM_10_	Retrospective study n = 203,562	Number of exposed were not specified. Exposure cut-offs of CO in II–IV Q: 0.8–1.3 μg/m^3^	Number of nonexposed were not specified. Exposure cut-offs of CO in I Q: 0.2–0.4μg/m^3^	NO_x_, NO_2_, CO, PM_2.5_, PM_10_ is related with increased risk of SGA infant. Small statistically significant association was observed for PM_10_ and SGA, particularly with exposure in the first and third trimesters. Similar effects on SGA were also found for NO_2_, PM_2.5_, and CO in later pregnancy, but no overall increased risk was observed.
da Silva et al. (2014) [59]	2004–2005 Mato Grosso, Brazil Pollutants: PM_2.5_ and CO	Retrospective study n = 6642	Number of exposed were not specified. Exposure with CO in II–IV Q: Cut-off point of exposure not specified in study.	Number of nonexposed were not specified. Exposure with CO in I Q: Cut-off point of exposure not specified in study.	Second trimester exposure (IV Q) to CO (aOR 1.49, 95%CI: 1.03–2.14) is related to increased risk of TLBW.
Le et al. (2012) [64]	1990–2001 Detroit, Michigan, USA Pollutants: CO, NO_2_, PM_10_ and O_3_	Retrospective study n = 164,905	Number of exposed were not specified. Exposure cut-offs of CO in II–IV Q: >0.75 ppm	Number of nonexposed were not specified. Exposure cut-offs of CO in I Q: <0.75 ppm	SGA was associated with CO exposure (aOR 1.14, 95%CI 1.02–1.27).
Nascimento and Moreira (2009) [73]	2001 São José dos Campos, Brazil Pollutants: SO_2_, O_3_ and PM_10_	Retrospective study n = 2529	Number of exposed were not specified. Exposure cut-offs of SO_2_ in II–IV Q: Cut-off point of exposure not specified in study.	Number of nonexposed were not specified. Exposure cut-offs of SO_2_ in I Q: Cut-off point of exposure not specified in study.	LBW was significantly associated with SO_2_ exposure in the II and III Q (aOR 1.30, 95%CI: 1.02–1.65).
Dugandzic et al. (2006) [77]	1988–2000 Nova Scotia Atlee, Canada Pollutants: PM_10_, SO_2_ and O_3_	Retrospective study n = 74,284	Number of exposed were not specified. Exposure cut-offs of SO_2_ in II–IV Q: 7–38 ppb	Number of nonexposed were not specified. Exposure cut-offs of SO_2_ in I Q: < 7 ppb	SO_2_ exposure during the I trimester is related with TLBW (RR 1.36, 95%CI: 1.04–1.78).
Wilhelm and Ritz (2005) [78]	1994–2000 South Coast Air Basin, Los Angeles, USA Pollutants: CO, PM_10_, PM_2.5_, O_3_ and NO_2_	Retrospective study n = 136,134	Number of exposed were not specified. Exposure cut-offs of SO_2_ in II–IV Q: II–III Q (0,91–1.82 pphm), IV Q CO (>1.82 pphm)	Number of nonexposed were not specified. Exposure cut-offs of SO_2_ in I Q: <0.91 pphm	IV Q CO exposures increase 36% in risk for in third-trimester pregnancy of developing TLBW.
Lin et al. (2004) [79]	1995–1997 Taipei and Kaohsiung, Taiwan Pollutants: SO_2_, PM_10_, CO, O_3_ and NO_2_	Retrospective study n = 31,530 (Kaohsiung) n = 60,758 (Taipei)	31,530 pregnant women from Kaohsiung exposed with mean concentration of CO (4.8–11.7 ppm)	60,758 pregnant women from Taipei exposed with mean concentration of CO (0.7–1.4 ppm)	Higher exposure of SO_2_, PM_10_, CO, O_3_, NO_2_ in Kaohsiung leads to 13% higher TLBW occurrence than lower exposure in Taipei (OR 1.13, 95%CI: 1.03–1.24).
Lin et al. (2004) [80]	1995–1997 Taipei and Kaohsiung, Taiwan Pollutants: SO_2_, PM_10_, CO, O_3_ and NO_2_	Retrospective study n = 92,288	Number of exposed were not specified. Exposure cut-offs of CO SO_2_ in II–IV Q: SO_2_ (>7.1 ppb) CO (>1.3 ppm)	Number of nonexposed were not specified. Exposure cut-offs of CO SO_2_ in I Q: SO_2_ (<7.1 ppb) CO (<1.3 ppm)	﻿Exposure to >12.4 ppb of SO_2_ in the third trimester related to 20% higher risk (OR 1.2, 95%CI: 1.01–1.41) of TLBW then exposure to <6.8 ppb (OR 1.20, 95%CI: 1.01–1.41). No associations were observed between PM_10_, CO, O_3_, or NO_2_ exposure and TLBW occurrence.
Lee et al. (2003) [81]	1996–1998 Seoul, Korea Pollutants: CO, PM_10_, SO_2_ and NO_2_	Retrospective study n = 388,105	Number of exposed were not specified. Exposure cut-offs of CO SO_2_ in II–IV Q: CO (0.9–3.4 ppm) SO_2_ (6.8–46.0 ppb)	Number of nonexposed were not specified. Exposure cut-offs of CO SO_2_ in I Q: CO (0.4–0.9 ppm) SO_2_ (3.0–6.8 ppb)	First-trimester CO exposure increased the risk for TLBW (aOR 1.04, 95%CI: 1.01–1.07), as did second-trimester exposure to SO_2_ (aOR 1.06, 95%CI: 1.02–1.11). CO, PM_10_, SO_2_ and NO_2_ during 1–2 trimesters were related with TLBW.
Yang et al. (2003) [82]	1995–1997 Kaohsiung, Taiwan Pollutants: SO_2_ and PM_10_	Retrospective study n = 13,396	Number of exposed were not specified. Exposure cut-offs of SO_2_ in II–III T: II T (26.02–36.07 μg/m^3^) III T (>36.07 μg/m^3^)	Number of nonexposed were not specified. Exposure cut-offs of SO_2_ in I T < 26.02 μg/m^3^	I trimester exposure of SO_2_ lead to reduced TBW (OR 18.1, 95%CI: 1.88–34.34).
Maroziene and Grazuleviciene (2002) [83]	1998 Kaunas, Lithuania Pollutants: Formaldehyde	Epidemiological study n = 3988	Number of exposed were not specified. Exposure with formaldehyde from II–III T. Tertiles of exposure cut-offs not specified in study.	Number of nonexposed were not specified. Exposure with formaldehyde in I T. Tertiles of exposure cut-offs not specified in study.	Formaldehyde exposure is related with TLBW in II T (aOR 1.86, 95%CI: 1.10–3.16) and in III T (aOR 1.84, 95%CI: 1.12–3.03). Most meaningful impact was observed in I trimester.
Chen et al. (2002) [84]	1991–1999 Nevada, USA Pollutants: PM_10_, CO and O_3_	Retrospective study n = 39,338	32,683 pregnant women exposed with CO at the third trimester (>0.62 ppm)	3622 pregnant women with low exposure to CO at the third trimester (<0.62 ppm)	CO and O_3_ were found not to be related to birth weight.
Vassilev et al. (2001) [85]	1990–1991 New Jersey, USA Pollutants: POM–polycyclic organic matter	Retrospective study n =199,474	132,484 pregnant women exposed with II–III T POM. Tertiles of exposure cut-offs: II T POM (0.27–0.61 μg/m^3^) III T POM (0.61–2.8 μg/m^3^)	66,990 pregnant women exposed with I T POM. Tertiles of exposure cut-offs: I T POM (0.04–0.27 μg/m^3^)	III T POM exposure is related with SGA (aOR 1.22, 95%CI: 1.17–1.27).
Lin et al. (2001) [86]	1993–1996 Lin-Yuan and Taicei, Taiwan Pollutants: SO_2_, NO_2_, PM_10_, SO_4_^2−^, NH_4_^+^ and NO_3_^−^	Retrospective study n = 2545	1677 pregnant women from Lin-Yuan municipality exposed with SO_2_, NO_2_, PM_10_, SO_4_^2−^, NH_4_^+^, NO_3_^−^ in II–IV Q: SO_2_ (6.0 ± 2.9 ppb) SO_4_^2−^ (120.2 ± 1.2 nmol/m^3^) NH_4_^+^ (136.1 ± 4.0 nmol/m^3^)	868 pregnant women from Taicei municipality exposed with SO_2_, NO_2_, PM_10_, SO_4_^2−^, NH_4_^+^, NO_3_^−^ in I Q: SO_2_ (1.9 ± 2.3 ppb) SO_4_^2−^ (91.4 ± 1.4 nmol/m^3^) NH^4^ (69.0 ± 3.1 nmol/m^3^)	Higher exposure of SO_2_, NO_2_, PM_10_, SO_4_^2−^, NO_3_^−^, petrochemical municipality in Lin-Yuan leads to 3.22% TLBW occurrence in comparison to lower exposure in control municipality Taicei which lead to 1.84% TLBW occurrence. Exposure to NH_4_^+^ influenced TLBW (aOR 1.77, 95%CI: 1.002–3.12).
Maisonet et al. (2001) [87]	1994–1996 Boston, Hartford, Philadelphia, Pittsburgh; Springfield, and Washington, USA Pollutants: CO, PM_10_ and SO_2_	Retrospective study n = 89,557	Number of exposed were not specified. Exposure cut-offs of CO and SO_2_ in II–IV Q: CO (0.93–1.5 ppm) SO_2_ (7.1–18.5 µg/m^3^)	Number of nonexposed were not specified. Exposure cut-offs of CO and SO_2_ in I Q: CO (<0.93 ppm) SO_2_ (<7.1 µg/m^3^)	SO_2_ and CO are related with TLBW. CO in third trimester (aOR 1.31, 95%CI: 1.06–1.62) and SO_2_ in second trimester within: II Q (aOR 1.21, 95%CI: 1.07–1.37), III Q (aOR 1.20, 95%CI: 1.08–1.35) IV Q (aOR 1.21, 95%CI: 1.03–1.43)
Ritz and Yu (1999) [88]	1989–1993 Los Angeles, USA Pollutant: CO	Retrospective study n = 125,573	62,787 pregnant women exposed with CO above the median. Exposure above median of 2.2–6.7 ppm CO	62,786 pregnant women exposed with CO below the median. Exposure blow median of 0.65–2.1 ppm CO	Exposure to (>5.5 ppm CO) during the third trimester is associated with TLBW (OR 1.22, 95%CI: 1.03–1.44)
Gražulevičienė et al. (1998) [89]	1994 Kaunas, Lithuania Pollutant: Formaldehyde	Retrospective study n = 4290	934 pregnant women exposed with formaldehyde >3.5 μg/m^3^ and 442 pregnant women exposed with O_3_ >30 μg/m^3^	3356 pregnant women exposed with formaldehyde <3.5 μg/m^3^ and 3848 pregnant women exposed with O_3_ <30 μg/m^3^	No associations were observed between formaldehyde and O_3_ exposure and SGA or TLBW occurrence.
Alderman et al. (1987) [90]	1975–1983 Colorado Department of Health, USA Pollutant: CO	Retrospective study, n = 2800	800 pregnant women exposed with CO form second quintile to fifth quintile. Number of women in each quintile is not specified. Quintiles exposure cut-offs of CO: II Q (1–2 ppm) III Q (2–3 ppm) IV Q (3–4 ppm) V Q (>4 ppm)	198 pregnant women exposed with CO in first quintile. I Quintile exposure cut-off of CO: <1 ppm	No significant association was observed between CO exposure and SGA or TLBW occurrence (OR 1.3, 95%CI: 1.0–1.7) for 2–4 ppm CO.

## Data Availability

Not applicable.

## Supplementary Materials

The following supporting information can be downloaded at: https://www.mdpi.com/article/10.3390/healthcare12121176/s1, Supplementary Table S1: PRISMA checklist; Supplementary Table S2: Newcastle–Ottawa risk bias score of the included studies.

## References

[1] Kajdy A., Feduniw S., Modzelewski J., Sys D., Filipecka-Tyczka D., Muzyka-Placzyńska K., Kiczmer P., Grabowski B., Rabijewski M. (2021). Growth Abnormalities as a Risk Factor of Adverse Neonatal Outcome in Hypertensive Pregnancies—A Single-Center Retrospective Cohort Study. Children.

[2] Beune I.M., Bloomfield F.H., Ganzevoort W., Embleton N.D., Rozance P.J., van Wassenaer-Leemhuis A.G., Wynia K., Gordijn S.J. (2018). Consensus Based Definition of Growth Restriction in the Newborn. J. Pediatr..

[3] Kajdy A., Sys D., Modzelewski J., Bogusławska J., Cymbaluk-Płoska A., Kwiatkowska E., Bednarek-Jędrzejek M., Borowski D., Stefańska K., Rabijewski M. (2023). Evidence of Placental Aging in Late SGA, Fetal Growth Restriction and Stillbirth—A Systematic Review. Biomedicines.

[4] Figueras F., Gratacós E. (2014). Update on the Diagnosis and Classification of Fetal Growth Restriction and Proposal of a Stage-Based Management Protocol. Fetal Diagn. Ther..

[5] Kajdy A., Modzelewski J., Jakubiak M., Pokropek A., Rabijewski M. (2019). Effect of Antenatal Detection of Small-for-Gestational-Age Newborns in a Risk Stratified Retrospective Cohort. PLoS ONE.

[6] American College of Obstetricians and Gynecologists (2013). Practice Bulletin No. 134. Obstet. Gynecol..

[7] Lausman A., Kingdom J., Gagnon R., Basso M., Bos H., Crane J., Davies G., Delisle M.F., Hudon L., Menticoglou S. (2013). Intrauterine Growth Restriction: Screening, Diagnosis, And Management. J. Obstet. Gynaecol. Can..

[8] Kajdy A., Modzelewski J., Cymbaluk-Płoska A., Kwiatkowska E., Bednarek-Jędrzejek M., Borowski D., Stefańska K., Rabijewski M., Torbé A., Kwiatkowski S. (2021). Molecular Pathways of Cellular Senescence and Placental Aging in Late Fetal Growth Restriction and Stillbirth. Int. J. Mol. Sci..

[9] Zhang Q., Zhang Z.C., He X.Y., Liu Z.M., Wei G.H., Liu X. (2022). Maternal Smoking during Pregnancy and the Risk of Congenital Urogenital Malformations: A Systematic Review and Meta-Analysis. Front. Pediatr..

[10] Akter S., Islam M.R., Rahman M.M., Rouyard T., Nsashiyi R.S., Hossain F., Nakamura R. (2023). Evaluation of Population-Level Tobacco Control Interventions and Health Outcomes: A Systematic Review and Meta-Analysis. JAMA Netw. Open.

[11] Athanasiadou K.I., Paschou S.A., Papakonstantinou E., Vasileiou V., Kanouta F., Kazakou P., Stefanaki K., Kassi G.N., Psaltopoulou T., Goulis D.G. (2023). Smoking during Pregnancy and Gestational Diabetes Mellitus: A Systematic Review and Meta-Analysis. Endocrine.

[12] Health Effects Institute Institute for Health Metrics and Evaluation’s Global Burden of Desease Project State of Global Air 2020. https://www.stateofglobalair.org/resources.

[13] WHO (2021). WHO Global Air Quality Guidelines: Particulate Matter (PM_2.5_ and PM_10_), Ozone, Nitrogen Dioxide, Sulfur Dioxide and Carbon Monoxide.

[14] Rehman A., Liu G., Yousaf B., Ijaz S., Irshad S., Cheema A.I., Riaz M.U., Ashraf A. (2023). Spectroscopic Fingerprinting, Pollution Characterization, and Health Risk Assessment of Potentially Toxic Metals from Urban Particulate Matter. Environ. Sci. Pollut. Res. Int..

[15] Nyadanu S.D., Dunne J., Tessema G.A., Mullins B., Kumi-Boateng B., Lee Bell M., Duko B., Pereira G. (2022). Prenatal Exposure to Ambient Air Pollution and Adverse Birth Outcomes: An Umbrella Review of 36 Systematic Reviews and Meta-Analyses. Environ. Pollut..

[16] Song S., Gao Z., Zhang X., Zhao X., Chang H., Zhang J., Yu Z., Huang C., Zhang H. (2023). Ambient Fine Particulate Matter and Pregnancy Outcomes: An Umbrella Review. Environ. Res..

[17] Shah P.S., Balkhair T., Knowledge Synthesis Group on Determinants of Preterm/LBW Births (2011). Air Pollution and Birth Outcomes: A Systematic Review. Environ. Int..

[18] Simoncic V., Enaux C., Deguen S., Kihal-Talantikite W. (2020). Adverse Birth Outcomes Related to NO_2_ and PM Exposure: European Systematic Review and Meta-Analysis. Int. J. Environ. Res. Public Health.

[19] Luo M., Liu T., Ma C., Fang J., Zhao Z., Wen Y., Xia Y., Zhao Y., Ji C. (2023). Household Polluting Cooking Fuels and Adverse Birth Outcomes: An Updated Systematic Review and Meta-Analysis. Front. Public Health.

[20] Page M.J., McKenzie J.E., Bossuyt P.M., Boutron I., Hoffmann T.C., Mulrow C.D., Shamseer L., Tetzlaff J.M., Akl E.A., Brennan S.E. (2021). The PRISMA 2020 Statement: An Updated Guideline for Reporting Systematic Reviews. BMJ.

[21] Wells G., Shea B., O’Connell D., Peterson J., Welch V., Losos M., Tugwell P. The Newcastle-Ottawa Scale (NOS) for Assessing the Quality of Nonrandomised Studies in Meta-Analyses. https://www.ohri.ca/programs/clinical_epidemiology/oxford.asp.

[22] Canto M.V., Guxens M., García-Altés A., López M.J., Marí-Dell’Olmo M., García-Pérez J., Ramis R. (2023). Air Pollution and Birth Outcomes: Health Impact and Economic Value Assessment in Spain. Int. J. Environ. Res. Public Health.

[23] Chen X., Chen S., Zhu Z., Luo J., Wang H., Wulayin M., Huang C., Zhao W., Wang Q. (2023). Identifying the Critical Windows and Joint Effects of Temperature and PM_2.5_ Exposure on Small for Gestational Age. Environ. Int..

[24] Mitku A.A., Zewotir T., North D., Jeena P., Asharam K., Muttoo S., Tularam H., Naidoo R.N. (2023). Impact of Ambient Air Pollution Exposure during Pregnancy on Adverse Birth Outcomes: Generalized Structural Equation Modeling Approach. BMC Public Health.

[25] Zhang F., Zhang X.X., Zhong Y., Zhu S., Zhao G., Zhang X.X., Li T., Zhang Y., Zhu W. (2023). Joint Exposure to Ambient Air Pollutants Might Elevate the Risk of Small for Gestational Age (SGA) Infants in Wuhan: Evidence From a Cross-Sectional Study. Int. J. Public Health.

[26] Zhou W., Ming X., Yang Y., Hu Y., He Z., Chen H., Li Y., Cheng J., Zhou X. (2023). Associations between Maternal Exposure to Ambient Air Pollution and Very Low Birth Weight: A Birth Cohort Study in Chongqing, China. Front. Public Health.

[27] Ahmad W.A., Nirel R., Golan R., Jolles M., Kloog I., Rotem R., Negev M., Koren G., Levine H. (2022). Mother-Level Random Effect in the Association between PM_2.5_ and Fetal Growth: A Population-Based Pregnancy Cohort. Environ. Res..

[28] Gan Q., Ye W., Zhao X., Teng Y., Mei S., Long Y., Ma J., Rehemutula R., Zhang X., Zeng F. (2022). Mediating Effects of Gut Microbiota in the Associations of Air Pollutants Exposure with Adverse Pregnancy Outcomes. Ecotoxicol. Environ. Saf..

[29] Gong X., Zhan F.B. (2022). A Method for Identifying Critical Time Windows of Maternal Air Pollution Exposures Associated with Low Birth Weight in Offspring Using Massive Geographic Data. Environ. Sci. Pollut. Res..

[30] Huang H.J., Yu Q.Y., Zheng T., Wang S.S., Yang X.J. (2022). Associations between Seasonal Ambient Air Pollution and Adverse Perinatal Outcomes: A Retrospective Cohort Study in Wenzhou, China. Environ. Sci. Pollut. Res..

[31] Rodríguez-Fernández A., Ramos-Castillo N., Ruiz-De la Fuente M., Parra-Flores J., Maury-Sintjago E. (2022). Association of Prematurity and Low Birth Weight with Gestational Exposure to PM_2.5_ and PM_10_ Particulate Matter in Chileans Newborns. Int. J. Environ. Res. Public Health.

[32] Shen Y., Wang C., Yu G., Meng X., Wang W., Kan H., Zhang J., Cai J. (2022). Associations of Ambient Fine Particulate Matter and Its Chemical Constituents with Birth Weight for Gestational Age in China: A Nationwide Survey. Environ. Sci. Technol..

[33] Zhu Z., Hu H., Benmarhnia T., Ren Z., Luo J., Zhao W., Chen S., Wu K., Zhang X., Wang L. (2022). Gestational PM_2.5_ Exposure May Increase the Risk of Small for Gestational Age through Maternal Blood Pressure and Hemoglobin: A Mediation Analysis Based on a Prospective Cohort in China, 2014–2018. Ecotoxicol. Environ. Saf..

[34] Chen J., Li P.H., Fan H., Li C., Zhang Y., Ju D., Deng F., Guo X., Guo L., Wu S. (2022). Weekly-Specific Ambient Fine Particular Matter Exposures before and during Pregnancy Were Associated with Risks of Small for Gestational Age and Large for Gestational Age: Results from Project ELEFANT. Int. J. Epidemiol..

[35] Chen Y., Hodgson S., Gulliver J., Granell R., Henderson A.J., Cai Y., Hansell A.L. (2021). Trimester Effects of Source-Specific PM_10_ on Birth Weight Outcomes in the Avon Longitudinal Study of Parents and Children (ALSPAC). Environ. Health Glob. Access Sci. Source.

[36] Shang L., Huang L., Yang L., Leng L., Qi C., Xie G., Wang R., Guo L., Yang W., Chung M.C. (2021). Impact of Air Pollution Exposure during Various Periods of Pregnancy on Term Birth Weight: A Large-Sample, Retrospective Population-Based Cohort Study. Environ. Sci. Pollut. Res..

[37] Wang Q., Miao H., Warren J.L., Ren M., Benmarhnia T., Knibbs L.D., Zhang H., Zhao Q., Huang C. (2021). Association of Maternal Ozone Exposure with Term Low Birth Weight and Susceptible Window Identification. Environ. Int..

[38] Bergstra A.D., Brunekreef B., Burdorf A. (2021). The Influence of Industry-Related Air Pollution on Birth Outcomes in an Industrialized Area. Environ. Pollut..

[39] Wojtyla C., Zielinska K., Wojtyla-Buciora P., Panek G. (2020). Prenatal Fine Particulate Matter (PM_2.5_) Exposure and Pregnancy Outcomes—Analysis of Term Pregnancies in Poland. Int. J. Environ. Res. Public Health.

[40] Tapia V.L., Vasquez B.V., Vu B., Liu Y., Steenland K., Gonzales G.F. (2020). Association between Maternal Exposure to Particulate Matter (PM_2.5_) and Adverse Pregnancy Outcomes in Lima, Peru. J. Expo. Sci. Environ. Epidemiol..

[41] Enders C., Pearson D., Harley K., Ebisu K. (2019). Exposure to Coarse Particulate Matter during Gestation and Term Low Birthweight in California: Variation in Exposure and Risk across Region and Socioeconomic Subgroup. Sci. Total Environ..

[42] Kim Y.J., Song I.G., Kim K.N., Kim M.S., Chung S.H., Choi Y.S., Bae C.W. (2019). Maternal Exposure to Particulate Matter during Pregnancy and Adverse Birth Outcomes in the Republic of Korea. Int. J. Environ. Res. Public Health.

[43] Nobles C.J., Grantz K.L., Liu D., Williams A., Ouidir M., Seeni I., Sherman S., Mendola P. (2019). Ambient Air Pollution and Fetal Growth Restriction: Physician Diagnosis of Fetal Growth Restriction versus Population-Based Small-for-Gestational Age. Sci. Total Environ..

[44] Percy Z., DeFranco E., Xu F., Hall E.S., Haynes E.N., Jones D., Muglia L.J., Chen A. (2019). Trimester Specific PM_2.5_ Exposure and Fetal Growth in Ohio, 2007–2010. Environ. Res..

[45] Gong X., Lin Y., Bell M.L., Zhan F.B. (2018). Associations between Maternal Residential Proximity to Air Emissions from Industrial Facilities and Low Birth Weight in Texas, USA. Environ. Int..

[46] Wu H., Jiang B., Geng X., Zhu P., Liu Z., Cui L., Yang L. (2018). Exposure to Fine Particulate Matter during Pregnancy and Risk of Term Low Birth Weight in Jinan, China, 2014–2016. Int. J. Hyg. Environ. Health.

[47] Fernando Costa Nascimento L., Blanco Machin A., Antonio Almeida Dos Santos D. (2017). Existem Diferenças No Peso Ao Nascer de Acordo Com Sexo e Associações Com Exposição Materna a Poluentes Do Ar? Estudo de Coorte. Sao Paulo Med. J..

[48] Dedele A., Grazuleviciene R., Miskinyte A. (2017). Individual Exposure to Nitrogen Dioxide and Adverse Pregnancy Outcomes in Kaunas Study. Int. J. Environ. Health Res..

[49] Capobussi M., Tettamanti R., Marcolin L., Piovesan L., Bronzin S., Gattoni M.E., Polloni I., Sabatino G., Tersalvi C.A., Auxilia F. (2016). Air Pollution Impact on Pregnancy Outcomes in Como, Italy. J. Occup. Environ. Med..

[50] Stieb D.M., Chen L., Hystad P., Beckerman B.S., Jerrett M., Tjepkema M., Crouse D.L., Omariba D.W., Peters P.A., van Donkelaar A. (2016). A National Study of the Association between Traffic-Related Air Pollution and Adverse Pregnancy Outcomes in Canada, 1999–2008. Environ. Res..

[51] Stieb D.M., Chen L., Beckerman B.S., Jerrett M., Crouse D.L., Omariba D.W.R., Peters P.A., Van Donkelaar A., Martin R.V., Burnett R.T. (2016). Associations of Pregnancy Outcomes and PM_2.5_ in a National Canadian Study. Environ. Health Perspect..

[52] Lavigne E., Yasseen A.S., Stieb D.M., Hystad P., van Donkelaar A., Martin R.V., Brook J.R., Crouse D.L., Burnett R.T., Chen H. (2016). Ambient Air Pollution and Adverse Birth Outcomes: Differences by Maternal Comorbidities. Environ. Res..

[53] Brown J.M., Harris G., Pantea C., Hwang S.A., Talbot T.O. (2015). Linking Air Pollution Data and Adverse Birth Outcomes: Environmental Public Health Tracking in New York State. J. Public Health Manag. Pract..

[54] Poirier A., Dodds L., Dummer T., Rainham D., Maguire B., Johnson M. (2015). Maternal Exposure to Air Pollution and Adverse Birth Outcomes in Halifax, Nova Scotia. J. Occup. Environ. Med..

[55] Twum C., Zhu J., Wei Y. (2015). Maternal Exposure to Ambient PM_2.5_ and Term Low Birthweight in the State of Georgia. Int. J. Environ. Health Res..

[56] Habermann M., Gouveia N. (2014). Socioeconomic Position and Low Birth Weight among Mothers Exposed to Traffic-Related Air Pollution. PLoS ONE.

[57] Hannam K., McNamee R., Baker P., Sibley C., Agius R. (2014). Air Pollution Exposure and Adverse Pregnancy Outcomes in a Large UK Birth Cohort: Use of a Novel Spatio-Temporal Modelling Technique. Scand. J. Work Environ. Health.

[58] Vinikoor-Imler L.C., Davis J.A., Meyer R.E., Messer L.C., Luben T.J. (2014). Associations between Prenatal Exposure to Air Pollution, Small for Gestational Age, and Term Low Birthweight in a State-Wide Birth Cohort. Environ. Res..

[59] da Silva A.M.C., Moi G.P., Mattos I.E., de Souza Hacon S. (2014). Low Birth Weight at Term and the Presence of Fine Particulate Matter and Carbon Monoxide in the Brazilian Amazon: A Population-Based Retrospective Cohort Study. BMC Pregnancy Childbirth.

[60] Hyder A., Lee H.J., Ebisu K., Koutrakis P., Belanger K., Bell M.L. (2014). PM_2.5_ Exposure and Birth Outcomes. Use of Satellite- and Monitor-Based Data. Epidemiology.

[61] Candela S., Ranzi A., Bonvicini L., Baldacchini F., Marzaroli P., Evangelista A., Luberto F., Carretta E., Angelini P., Sterrantino A.F. (2013). Air Pollution from Incinerators and Reproductive Outcomes: A Multisite Study. Epidemiology.

[62] Olsson D., Mogren I., Forsberg B. (2013). Air Pollution Exposure in Early Pregnancy and Adverse Pregnancy Outcomes: A Register-Based Cohort Study. BMJ. Open.

[63] Sathyanarayana S., Zhou C., Rudra C.B., Gould T., Larson T., Koenig J., Karr C.J. (2013). Prenatal Ambient Air Pollution Exposure and Small for Gestational Age Birth in the Puget Sound Air Basin. Air Qual. Atmos. Health.

[64] Le H.Q., Batterman S.A., Wirth J.J., Wahl R.L., Hoggatt K.J., Sadeghnejad A., Hultin M.L., Depa M. (2012). Air Pollutant Exposure and Preterm and Term Small-for-Gestational-Age Births in Detroit, Michigan: Long-Term Trends and Associations. Environ. Int..

[65] van den Hooven E.H., Pierik F.H., de Kluizenaar Y., Willemsen S.P., Hofman A., van Ratingen S.W., Zandveld P.Y.J., Mackenbach J.P., Steegers E.A.P., Miedema H.M.E. (2012). Air Pollution Exposure during Pregnancy, Ultrasound Measures of Fetal Growth, and Adverse Birth Outcomes: A Prospective Cohort Study. Environ. Health Perspect..

[66] Salihu H.M., August E.M., Mbah A.K., Alio A.P., De Cuba R., Jaward F.M., Berry E. (2012). Lo Effectiveness of a Federal Healthy Start Program in Reducing the Impact of Particulate Air Pollutants on Feto-Infant Morbidity Outcomes. Matern. Child Health J..

[67] Salihu H.M., Ghaji N., Mbah A.K., Alio A.P., August E.M., Boubakari I. (2012). Particulate Pollutants and Racial/Ethnic Disparity in Feto-Infant Morbidity Outcomes. Matern. Child Health J..

[68] Malmqvist E., Rignell-Hydbom A., Tinnerberg H., Björk J., Stroh E., Jakobsson K., Rittner R., Rylander L. (2011). Maternal Exposure to Air Pollution and Birth Outcomes. Environ. Health Perspect..

[69] Kashima S., Naruse H., Yorifuji T., Ohki S., Murakoshi T., Takao S., Tsuda T., Doi H. (2011). Residential Proximity to Heavy Traffic and Birth Weight in Shizuoka, Japan. Environ. Res..

[70] Gehring U., Van Eijsden M., Dijkema M.B.A., Van Der Wal M.F., Fischer P., Brunekreef B. (2011). Traffic-Related Air Pollution and Pregnancy Outcomes in the Dutch ABCD Birth Cohort Study. Occup. Environ. Med..

[71] Madsen C., Gehring U., Erik Walker S., Brunekreef B., Stigum H., Næss Ø., Nafstad P. (2010). Ambient Air Pollution Exposure, Residential Mobility and Term Birth Weight in Oslo, Norway. Environ. Res..

[72] Ballester F., Estarlich M., Iñiguez C., Llop S., Ramón R., Esplugues A., Lacasaña M., Rebagliato M. (2010). Air Pollution Exposure during Pregnancy and Reduced Birth Size: A Prospective Birth Cohort Study in Valencia, Spain. Environ. Health A Glob. Access Sci. Source.

[73] Nascimento L.F.C., Moreira D.A. (2009). Are Environmental Pollutants Risk Factors for Low Birth Weight? TT—Os Poluentes Ambientais São Fatores de Risco Para o Baixo Peso Ao Nascer?. Cad. Saude Publica.

[74] Brauer M., Lencar C., Tamburic L., Koehoorn M., Demers P., Karr C. (2008). A Cohort Study of Traffic-Related Air Pollution Impacts on Birth Outcomes. Environ. Health Perspect..

[75] Hansen C., Neller A., Williams G., Simpson R. (2007). Low Levels of Ambient Air Pollution during Pregnancy and Fetal Growth among Term Neonates in Brisbane, Australia. Environ. Res..

[76] Kim O.J., Ha E.H., Kim B.M., Seo J.H., Park H.S., Jung W.J., Lee B.E., Suh Y.J., Kim Y.J., Lee J.T. (2007). PM_10_ and Pregnancy Outcomes: A Hospital-Based Cohort Study of Pregnant Women in Seoul. J. Occup. Environ. Med..

[77] Dugandzic R., Dodds L., Stieb D., Smith-Doiron M. (2006). The Association between Low Level Exposures to Ambient Air Pollution and Term Low Birth Weight: A Retrospective Cohort Study. Environ. Health A Glob. Access Sci. Source.

[78] Wilhelm M., Ritz B. (2005). Local Variations in CO and Particulate Air Pollution and Adverse Birth Outcomes in Los Angeles County, California, USA. Environ. Health Perspect..

[79] Lin C.M., Li C.Y., Mao I.F. (2004). Increased Risks of Term Low-Birth-Weight Infants in a Petrochemical Industrial City with High Air Pollution Levels. Arch. Environ. Health.

[80] Lin C.M., Li C.Y., Yang G.Y., Mao I.F. (2004). Association between Maternal Exposure to Elevated Ambient Sulfur Dioxide during Pregnancy and Term Low Birth Weight. Environ. Res..

[81] Lee B.E., Ha E.H., Park H.S., Kim Y.J., Hong Y.C., Kim H., Lee J.T. (2003). Exposure to Air Pollution during Different Gestational Phases Contributes to Risks of Low Birth Weight. Human Reprod..

[82] Yang C.Y., Tseng Y.T., Chang C.C. (2003). Effects of Air Pollution on Birth Weight among Children Born between 1995 and 1997 in Kaohsiung, Taiwan. J. Toxicol. Environ. Health Part A.

[83] Maroziene L., Grazuleviciene R. (2002). Maternal Exposure to Low-Level Air Pollution and Pregnancy Outcomes: A Population-Based Study. Environ. Health.

[84] Chen L., Yang W., Jennison B.L., Goodrich A., Omaye S.T. (2002). Air Pollution and Birth Weight in Northern Nevada, 1991–1999. Inhal. Toxicol..

[85] Vassilev Z.P., Robson M.G., Klotz J.B. (2001). Outdoor Exposure to Airborne Polycyclic Organic Matter and Adverse Reproductive Outcomes: A Pilot Study. Am. J. Ind. Med..

[86] Lin M.C., Yu H.S., Tsai S.S., Cheng B.H., Hsu T.Y., Wu T.N., Yang C.Y. (2001). Adverse Pregnancy Outcome in a Petrochemical Polluted Area in Taiwan. J. Toxicol. Environ. Health Part A.

[87] Maisonet M., Bush T.J., Correa A., Jaakkola J.J.K. (2001). Relation between Ambient Air Pollution and Low Birth Weight in the Northeastern United States. Environ. Health Perspect..

[88] Ritz B., Yu F. (1999). The Effect of Ambient Carbon Monoxide on Low Birth Weight among Children Born in Southern California between 1989 and 1993. Environ. Health Perspect..

[89] Gražulevičiené R., Dulskiené V., Vencloviené J. (1998). Formaldehyde Exposure and Low Birth Weight Incidence. J. Occup. Health.

[90] Alderman B.W., Baron A.E., Savitz D.A. (1987). Maternal Exposure to Neighborhood Carbon Monoxide and Risk of Low Infant Birth Weight. Public Health Rep..

[91] Ghio A.J., Kim C., Devlin R.B. (1999). Concentrated Ambient Air Particles Induce Mild Pulmonary Inflammation in Healthy Human Volunteers. Am. J. Respir. Crit. Care Med..

[92] Jiang Y., Van Nguyen T., Jin J., Yu Z.N., Song C.H., Chai O.H. (2023). Bergapten Ameliorates Combined Allergic Rhinitis and Asthma Syndrome after PM_2.5_ Exposure by Balancing Treg/Th17 Expression and Suppressing STAT3 and MAPK Activation in a Mouse Model. Biomed. Pharmacother..

[93] Liu J., Chen Y., Liu D., Ye F., Sun Q., Huang Q., Dong J., Pei T., He Y., Zhang Q. (2023). Prenatal Exposure to Particulate Matter and Term Low Birth Weight: Systematic Review and Meta-Analysis. Environ. Sci. Pollut. Res. Int..

[94] Gangwar R.S., Bevan G.H., Palanivel R., Das L., Rajagopalan S. (2020). Oxidative Stress Pathways of Air Pollution Mediated Toxicity: Recent Insights. Redox Biol..

[95] Checa J., Aran J.M. (2020). Reactive Oxygen Species: Drivers of Physiological and Pathological Processes. J. Inflamm. Res..

[96] Dick C.A.J., Singh P., Daniels M., Evansky P., Becker S., Gilmour M.I. (2003). Murine Pulmonary Inflammatory Responses Following Instillation of Size-Fractionated Ambient Particulate Matter. J. Toxicol. Environ. Health A.

[97] Simkhovich B., Kleinman M.T., Kloner R.A. (2008). Air Pollution and Cardiovascular Injury. Am. Coll. Cardiol. Found..

[98] Bearblock E., Aiken C.E., Burton G.J. (2021). Air Pollution and Pre-Eclampsia; Associations and Potential Mechanisms. Placenta.

[99] Kosinska-Kaczynska K., Malicka E., Szymusik I., Dera N., Pruc M., Feduniw S., Rafique Z., Szarpak L. (2023). The sFlt-1/PlGF Ratio in Pregnant Patients Affected by COVID-19. J. Clin. Med..

[100] Hettfleisch K., Carvalho M.A., Hoshida M.S., Pastro L.D.M., Saldiva S.R.D.M., Vieira S.E., Francisco R.P.V., Saldiva P.H.N., Bernardes L.S. (2021). Individual Exposure to Urban Air Pollution and Its Correlation with Placental Angiogenic Markers in the First Trimester of Pregnancy, in São Paulo, Brazil. Environ. Sci. Pollut. Res. Int..

[101] Arsalane K., Gosset P., Vanhee D., Voisin C., Hamid Q., Tonnel A.B., Wallaert B. (1995). Ozone Stimulates Synthesis of Inflammatory Cytokines by Alveolar Macrophages in Vitro. Am. J. Respir. Cell Mol. Biol..

[102] Beckerman B., Jerrett M., Brook J.R., Verma D.K., Arain M.A., Finkelstein M.M. (2008). Correlation of Nitrogen Dioxide with Other Traffic Pollutants near a Major Expressway. Atmos. Environ..

[103] Gentner D.R., Jathar S.H., Gordon T.D., Bahreini R., Day D.A., El Haddad I., Hayes P.L., Pieber S.M., Platt S.M., De Gouw J. (2017). Review of Urban Secondary Organic Aerosol Formation from Gasoline and Diesel Motor Vehicle Emissions. Environ. Sci. Technol..

[104] Peitzmeier C., Loschke C., Wiedenhaus H., Klemm O. (2017). Real-World Vehicle Emissions as Measured by in Situ Analysis of Exhaust Plumes. Environ. Sci. Pollut. Res..

[105] Hata H., Okada M., Yanai K., Kugata M., Hoshi J. (2022). Exhaust Emissions from Gasoline Vehicles after Parking Events Evaluated by Chassis Dynamometer Experiment and Chemical Kinetic Model of Three-Way Catalytic Converter. Sci. Total Environ..

[106] Mendoza-Ramirez J., Barraza-Villarreal A., Hernandez-Cadena L., de la Garza O.H., Sangrador J.L.T., Torres-Sanchez L.E., Cortez-Lugo M., Escamilla-Nuñez C., Sanin-Aguirre L.H., Romieu I. (2018). Prenatal Exposure to Nitrogen Oxides and Its Association with Birth Weight in a Cohort of Mexican Newborns from Morelos, Mexico. Ann. Glob. Health.

[107] Cyr A.R., Huckaby L.V., Shiva S.S., Zuckerbraun B.S. (2020). Nitric Oxide and Endothelial Dysfunction. Crit. Care Clin..

[108] Choi Y.-J., Cho J., Hong Y.-C., Lee D., Moon S., Park S.J., Lee K., Shin C.H., Lee Y.A., Kim B.-N. (2023). DNA Methylation Is Associated with Prenatal Exposure to Sulfur Dioxide and Childhood Attention-Deficit Hyperactivity Disorder Symptoms. Sci. Rep..

[109] Gozubuyuk A.A., Dag H., Kacar A., Karakurt Y., Arica V. (2017). Epidemiology, Pathophysiology, Clinical Evaluation, and Treatment of Carbon Monoxide Poisoning in Child, Infant, and Fetus. North Clin. Istanb..

[110] Chou K.J., Fisher J.L., Silver E.J. (2000). Characteristics and Outcome of Children with Carbon Monoxide Poisoning with and without Smoke Exposure Referred for Hyperbaric Oxygen Therapy. Pediatr. Emerg. Care.

[111] Perera F.P., Jedrychowski W., Rauh V., Whyatt R.M. (1999). Molecular Epidemiologic Research on the Effects of Environmental Pollutants on the Fetus. Environ. Health Perspect..

[112] Liu J., Dai Y., Yuan J., Li R., Hu Y., Su Y. (2023). Does Exposure to Air Pollution during Different Time Windows Affect Pregnancy Outcomes of in Vitro Fertilization Treatment? A Systematic Review and Meta-Analysis. Chemosphere.

[113] Bai W., Li Y., Niu Y., Ding Y., Yu X., Zhu B., Duan R., Duan H., Kou C., Li Y. (2020). Association between Ambient Air Pollution and Pregnancy Complications: A Systematic Review and Meta-Analysis of Cohort Studies. Environ. Res..

[114] Issah I., Duah M.S., Arko-Mensah J., Bawua S.A., Agyekum T.P., Fobil J.N. (2024). Assessing the Combined Effect of Multiple Metal Exposures on Pregnancy and Birth Outcomes: Methodological Insights in Systematic Review Research. MethodsX.

