# Supplementary material for: Does Exposure to Ambient Air Pollution Affect Gestational Age and Newborn Weight?—A Systematic Review
Source: Healthcare (Basel). 2024 Jun 11;12(12):1176. doi: 10.3390/healthcare12121176 (PMC11203000; doi:10.3390/healthcare12121176)
Supplement: Supplementary file 1 [file healthcare-12-01176-s001.zip › healthcare-3018772-S2.pdf]

Supplementary Table S2: Newcastle-Ottawa risk bias score of included studies.

	**Selection (A)**				**Comparability (B)**		**Outcome (C)**			
	**Exposed truly representative of avarage**	**Selection of non-exposed from the same community**	**Exposure of ascertained by secure record or interview**	**Demonstration of outcome of interest not present at the start of the study**	**Comparability of cohorts on the basis of the design or analysis**	**Study controls for other variables**	**Asssessment of outcomes**	**Follow uo long enough for outcome to occur**	**Adequacy of follow-up of cohorts**	**Score**
**Wojtyla et al. 2020**	1	1	1	1	1	1	1	1	0	**8**
**Enders et al. 2019**	1	1	1	1	0	1	0	1	0	**6**
**Kim et al. 2019**	1	1	1	1	0	1	0	1	0	**6**
**Nobles et al. 2019**	1	1	1	1	0	1	1	1	0	**7**
**Percy et al. (2019)**	1	1	1	1	1	1	1	1	0	**8**
**Gong et al. (2018)**	1	1	1	1	0	1	0	1	0	**6**
**Wu (2018)**	1	1	1	1	0	1	0	1	0	**6**
**Nascimento et al. (2017)**	1	1	1	1	0	1	0	1	0	**6**
**Capobussi et al. (2016)**	1	1	1	1	0	1	1	1	0	**7**
**Brown et al. (2015)**	1	1	1	1	0	1	1	1	0	**7**
**Poirier et al. (2015)**	1	1	1	1	0	1	0	1	0	**6**
**Twum et al. (2015)**	1	1	1	1	1	1	0	1	0	**7**
**Habermann et al. (2014)**	1	1	1	1	1	1	0	1	0	**7**
**Hannam et al. (2014)**	1	1	1	1	0	1	1	1	0	**7**

**Candela et al. (2013)**	1	1	1	1	1	1	1	1	0	**8**
**Olsson et al. (2013)**	1	1	1	1	0	1	1	1	0	**7**
**Le et al. (2012)**	1	1	1	1	0	1	1	1	0	**7**
**Salihu et al. (2012)**	1	1	1	1	1	1	1	1	0	**8**
**van den Hooven et al. (2012)**	1	1	1	1	1	1	1	1	1	**9**
**Malmqvist et al. (2011)**	1	1	1	1	1	1	1	1	0	**8**
**Salihu et al. (2011)**	1	1	1	1	1	1	1	1	0	**8**
**Madsen et al. (2010)**	1	1	1	1	1	1	1	1	0	**8**
**Nascimento and Moreira (2009)**	1	1	1	1	0	1	0	1	0	**6**
**Hansen et al. (2007)**	1	1	1	1	0	1	1	1	0	**7**
**Kim et al. (2007)**	1	1	1	1	0	1	1	1	1	**8**
**Dugandzic et al. (2006)**	1	1	1	1	0	1	0	1	0	**6**
**Wilhelm et al. (2005)**	1	1	1	1	0	1	0	1	0	**6**
**Lin et al. (2004)**	1	1	1	1	1	1	0	1	0	**7**
**Lee et al. (2003)**	1	1	1	1	0	1	0	1	0	**6**
**Yang et al. (2003)**	1	1	1	1	0	1	0	1	0	**6**

**Maroziene and Grazuleviciene (2002)**	1	1	1	1	0	1	0	1	0	**6**
**Chen et al. (2002)**	1	1	1	1	1	1	0	1	0	**7**
**Vassilev et al. (2001)**	1	1	1	1	1	1	1	1	0	**8**
**Lin et al. (2001)**	1	1	1	1	1	1	0	1	0	**7**
**Maisonet et al. (2001)**	1	1	1	1	0	1	0	1	0	**6**
**Ritz and Yu (1999)**	1	1	1	1	1	1	0	1	0	**7**
**Gražulevičienė et al. (1998)**	1	1	1	1	1	1	1	1	0	**8**
**Alderman et al. (1987)**	1	1	1	1	1	0	1	1	0	**7**
**Canto et al. (2023)**	1	1	1	1	1	1	1	1	0	**8**
**Chen et al. (2023)**	1	1	1	1	0	1	1	1	1	**8**
**Mitku et al. (2023)**	1	1	1	1	0	1	1	1	0	**7**
**(Zhang et al., 2023)**	1	1	1	1	0	1	1	1	0	**7**
**(Zhou et al., 2023)**	1	1	1	1	0	1	0	1	0	**6**
**(Ahmad et al., 2022)**	1	1	1	1	0	1	1	1	0	**7**
**(Gan et al., 2022)**	1	1	1	1	0	1	0	1	1	**7**
**(Gong and Zhan, 2022)**	1	1	1	1	1	1	0	1	0	**7**
**(Huang et al., 2022)**	1	1	1	1	0	1	0	1	0	**6**

**(Rodríguez-Fernández et al., 2022)**	1	1	1	1	0	1	0	1	0	**6**
**(Shen et al., 2022)**	1	1	1	1	0	1	1	1	0	**7**
**(Zhu et al., 2022)**	1	1	1	1	0	1	1	1	1	**8**
**(Chen et al., 2022)**	1	1	1	1	0	1	1	1	0	**7**
**(Chen et al., 2021)**	1	1	1	1	0	1	1	1	0	**7**
**(Shang et al., 2021)**	1	1	1	1	0	1	0	1	0	**6**
**(Wang et al., 2021)**	1	1	1	1	0	1	0	1	0	**6**
**(Bergstra et al., 2021)**	1	1	1	1	0	1	0	1	0	**6**
**(Tapia et al., 2020)**	1	1	1	1	0	1	1	1	0	**7**
**(Dedele et al., 2017)**	1	1	1	1	1	1	0	1	0	**7**
**(Stieb et al., 2016b)**	1	1	1	1	0	1	1	1	0	**7**
**(Stieb et al., 2016a)**	1	1	1	1	0	1	1	1	0	**7**
**(Lavigne et al., 2016)**	1	1	1	1	0	1	1	1	1	**8**
**(Vinikoor-Imler et al., 2014)**	1	1	1	1	0	1	1	1	0	**7**
**(da Silva et al., 2014)**	1	1	1	1	0	1	0	1	0	**6**
**(Hyder et al., 2014)**	1	1	1	1	0	1	1	1	0	**7**
**(Sathyanarayana et al., 2013)**	1	1	1	1	0	1	1	1	0	**7**
**(Kashima et al., 2011)**	1	1	1	1	0	1	1	1	0	**7**
**(Gehring et al., 2011)**	1	1	1	1	0	1	1	1	0	**7**

**(Ballester et al., 2010)**	1	1	1	1	0	1	1	1	0	**7**
**(Brauer et al., 2008)**	1	1	1	1	0	1	1	1	0	**7**
